# Transcriptional coregulator ZMIZ1 modulates estrogen responses that are essential for healthy endometrial function

**DOI:** 10.1172/JCI193212

**Published:** 2025-12-01

**Authors:** Sylvia C. Hewitt, Frank Orellana, Ryan M. Marquardt, MyeongJin Yi, Cynthia J. Willson, Mark Y. Chiang, Yong Song, Goutham Venkata Naga Davuluri, Christopher Day, Ramakrishna Kommagani, Joseph Rodriguez, Asgerally T. Fazleabas, John P. Lydon, Francesco J. DeMayo

**Affiliations:** 1 Pregnancy & Female Reproduction Group, Reproductive and Developmental Biology Lab, NIEHS, Research Triangle Park, North Carolina, USA.; 2 Inotiv-RTP, Durham, North Carolina, USA.; 3 Division of Hematology-Oncology, Department of Internal Medicine, University of Michigan School of Medicine, Ann Arbor, Michigan, USA.; 4 Department of Obstetrics, Gynecology & Reproductive Biology, Michigan State University, Grand Rapids, Michigan, USA.; 5 Department of Pathology & Immunology, Baylor College of Medicine, Houston, Texas, USA.; 6 Single Cell Dynamics Group, Epigenetics & RNA Biology Laboratory, NIEHS, Research Triangle Park, North Carolina, USA.; 7 Department of Molecular and Cellular Biology, Baylor College of Medicine, Houston, Texas, USA.

**Keywords:** Endocrinology, Reproductive biology, Reproductive biochemistry, Sex hormones, Transcription

## Abstract

Estrogen is a critical regulator of endometrial health. Aberrant estrogen stimulation can result in infertility, endometrial cancer, and endometriosis. Here, we identified *Zinc Finger MIZ-Type Containing 1* (*Zmiz1*) as a coregulator of uterine estrogen signaling. *ZMIZ1* is colocalized with an estrogen receptor α–binding (ESR1-binding) super enhancer. *ZMIZ1* mutations are found in endometrial cancer and its RNA levels trend toward reduction in endometrium of patients with endometriosis. *ZMIZ1* is dynamically expressed in human endometrial tissues during the menstrual cycle. Disrupting *ZMIZ1* in cultured human endometrial stromal cells resulted in impaired cell proliferation and decidual differentiation. Ablation of *Zmiz1* using the Pgr^Cre^ mouse (Zmiz1^d/d^) resulted in infertility and accelerated age-dependent uterine fibrosis. Zmiz1^d/d^ mice showed reduced ovulation and progesterone levels while maintaining normal serum prolactin during pregnancy. Uteri of Zmiz1^d/d^ mice were unable to undergo a hormonally induced decidual response, had decreased expression of stromal progesterone receptor (PGR) and decreased stromal and epithelial cell proliferation. Analysis of the transcriptome of Zmiz1^d/d^ mouse uteri showed decreased E2F, CCNA2, and FOXM1 signaling. Challenging ovariectomized Zmiz1^d/d^ mice with estrogen resulted in a decreased amplitude of some estrogen-regulated gene responses. Our findings demonstrate the importance of ZMIZ1 as an ESR1 coregulator in uterine biology and pathology.

## Graphical abstract

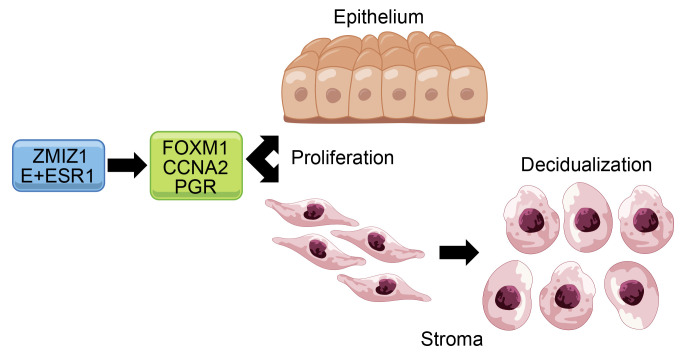

## Introduction

Healthy endometrial function is crucial not only for successful reproduction but also for the life-long general health and well being of women worldwide. In their lifetimes, most women will seek medical care, frequently for management of fertility, often to obtain contraception (99% of women in the USA) (1), or for care arising from endometrial dysfunction that contributes to infertility and pregnancy complications during reproductive years (2–4). Other common uterine dysfunctions include fibroids (60% of women) (5), issues related to sexual health or infections, uterine cancers (6), and endometriosis (7). Many endometrial health concerns onset with puberty, when the ovary begins to secrete the hormones estrogen and progesterone. Aberrant hormonal stimulation can result in endometrial hyperplasia and augment diseases such as endometrial cancer and endometriosis (8, 9). Even gestational exposures, as revealed by the unfortunate incident of “DES Daughters,” can greatly impact future health due to developmental defects on the uterus (10).

The ovarian hormones estrogen and progesterone act through their cognate receptors, estrogen receptor α (ESR1) and progesterone receptor (PGR), respectively. These receptors work within endometrial cells to regulate gene transcription by modifying chromatin and interacting with transcriptional regulators that modulate numerous intracellular and intercellular signaling pathways (11, 12). Hormone receptor–chromatin interactions at enhancer regions, located both proximal to and distal from coding genes, concentrate transcriptional machinery to modulate gene expression (12). Genes associated with super enhancers, enhancers exhibiting elevated enrichment of active enhancer histone modification, often encode mediators important to cell-type–specific development and function (13). Analysis of chromatin isolated from mouse uterine tissue revealed an ESR1-binding super enhancer at the *Zinc finger MIZ-type containing 1* (*Zmiz1*) gene (14). The location of *Zmiz1* at an ESR1 super enhancer may indicate the importance of this gene in ESR1 regulation of endometrial function.

*Zmiz1* encodes a 1,067 amino acid protein that, despite lacking DNA binding, exhibits characteristics of a transcriptional regulator, including a tetratricopeptide repeat (TPR) that interacts with NOTCH1 (15), a nuclear localization signal motif, an X-SPRING (extended SP-ring) zinc finger domain that interacts with AR, SMADs, and TP53 (15, 16), as well as extensive unstructured protein domains (intrinsically disordered regions), which are associated with transcriptional regulation activity (17, 18), including a transcriptional activation domain (TAD). ZMIZ1 modulates activity of multiple transcription factors, including AR, SMAD, NOTCH, TP53, and ESR1 (15, 16, 19, 20). Biologically, ZMIZ1, via its NOTCH1 coactivator activity, is involved in T cell development (21); ZMIZ1/NOTCH1 signaling also influences the development of acute T lymphoblastic leukemias (T-ALL) (22). ZMIZ1/NOTCH1 signaling impacts growth and invasiveness of tongue squamous cell carcinoma cells as well (23). ZMIZ1 is coexpressed with ESR1 in breast tumors and enhances the proliferation of breast cancer cells (20). Its roles in cancers suggest ZMIZ1 as a potential therapeutic target. Other biological functions are indicated by identification of GWAS *ZMIZ1* variants correlated with autoimmune disorders and type 2 diabetes risk (15, 24). A crucial role for ZMIZ1 in brain development was realized when a cohort of 19 individuals with neurodevelopmental issues were found to have *ZMIZ1* mutations (25). Subsequent work has characterized its importance in developing brain structures and has shown correlation between *ZMIZ1* mutations and autism as well (26). The expression and biological roles of ZMIZ1 have not been previously characterized in nondiseased uterine tissue; however, its location at an ESR1-binding super enhancer (14), and interaction with hormone receptors, AR in prostate cancer cells (27), and ESR1 in breast cancer cells, prompted consideration of potential roles for ZMIZ1 in endometrial health. Here, we investigate the role of ZMIZ1 in human endometrial stromal cells and the mouse uterus, as well as potential impacts on endometrial cancer and endometriosis, using human and mouse models.

## Results

### ZMIZ1 expression in human endometrium.

To investigate potential roles of ZMIZ1 in human endometrial function, we compared chromatin landscapes near mouse (14) and human *ZMIZ1* genes (Supplemental Figure 1; supplemental material available online with this article; https://doi.org/10.1172/JCI193212DS1). ESR1 ChIP seq from proliferative phase endometrial biopsies (28) reveals multiple peaks at the *ZMIZ1* gene as well as at distal regions 90 and 135 kb 5′ of the *ZMIZ1* transcription start site (TSS; Supplemental Figure 1A). Additionally, H3K27Ac ChIP seq of cultured human endometrial stromal cells (hESC) indicates the presence of this active enhancer mark coinciding with the ESR1-binding regions, showing that the *ZMIZ1* gene enhancers in mouse uterus and human endometrium are similar. HiC analysis of chromatin interactions in mouse uterus and hESC show interaction between these putative enhancers and the *ZMIZ1* gene (Supplemental Figure 1A).

**Figure 1:**
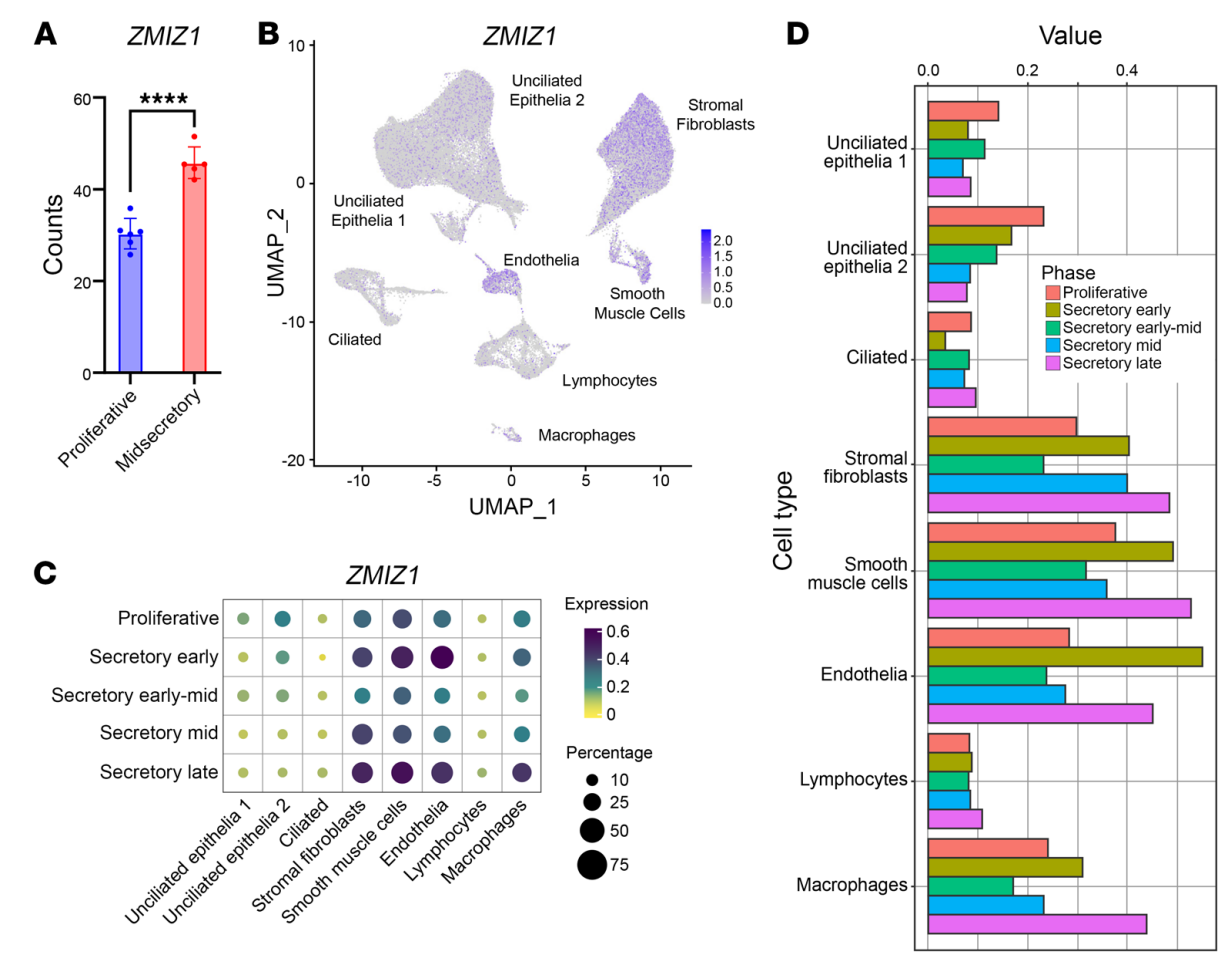
*ZMIZ1* expression in human endometrium. (**A**) *ZMIZ1* expression in RNA-seq datasets from proliferative or midsecretory endometrial biopsies (GSE132713; *n* = 6 proliferative, 5 midsecretory; *P* < 0.0001 by unpaired 2-tailed *t* test). (**B**) UMAP shows scRNA-seq *ZMIZ1* signal in human endometrial cells. (**C**) Bubble plot shows Expression level and percentage of cells of each cell type expressing *ZMIZ1* at multiple menstrual cycle stages. (**D**) Bar graph shows expression level of *ZMIZ1* in each cell type at each menstrual cycle stage.

**Figure 2:**
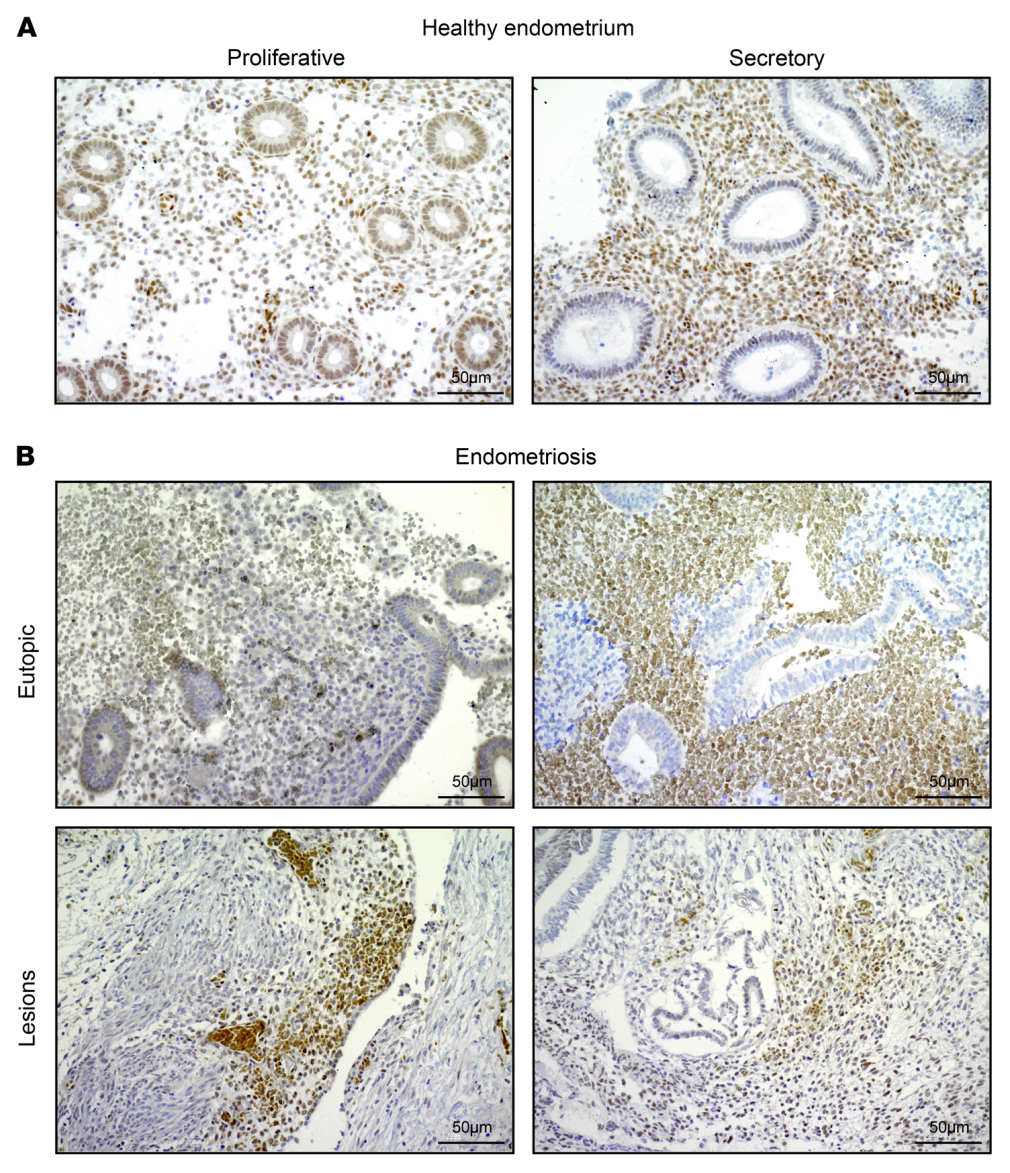
ZMIZ1 expression in human healthy and endometriosis endometrium and lesions. (**A**) ZMIZ1 IHC showing representative samples of proliferative (*n* = 5) and secretory (*n* = 5) phase endometrium from healthy controls. Scale bar: 50 μm. (**B**) ZMIZ1 IHC showing representative samples of eutopic endometrium (*n* = 9) and endometriotic lesions (*n* = 9) obtained from women with endometriosis. Scale bar: 50 μm. Cycle stage was not available.

**Figure 3:**
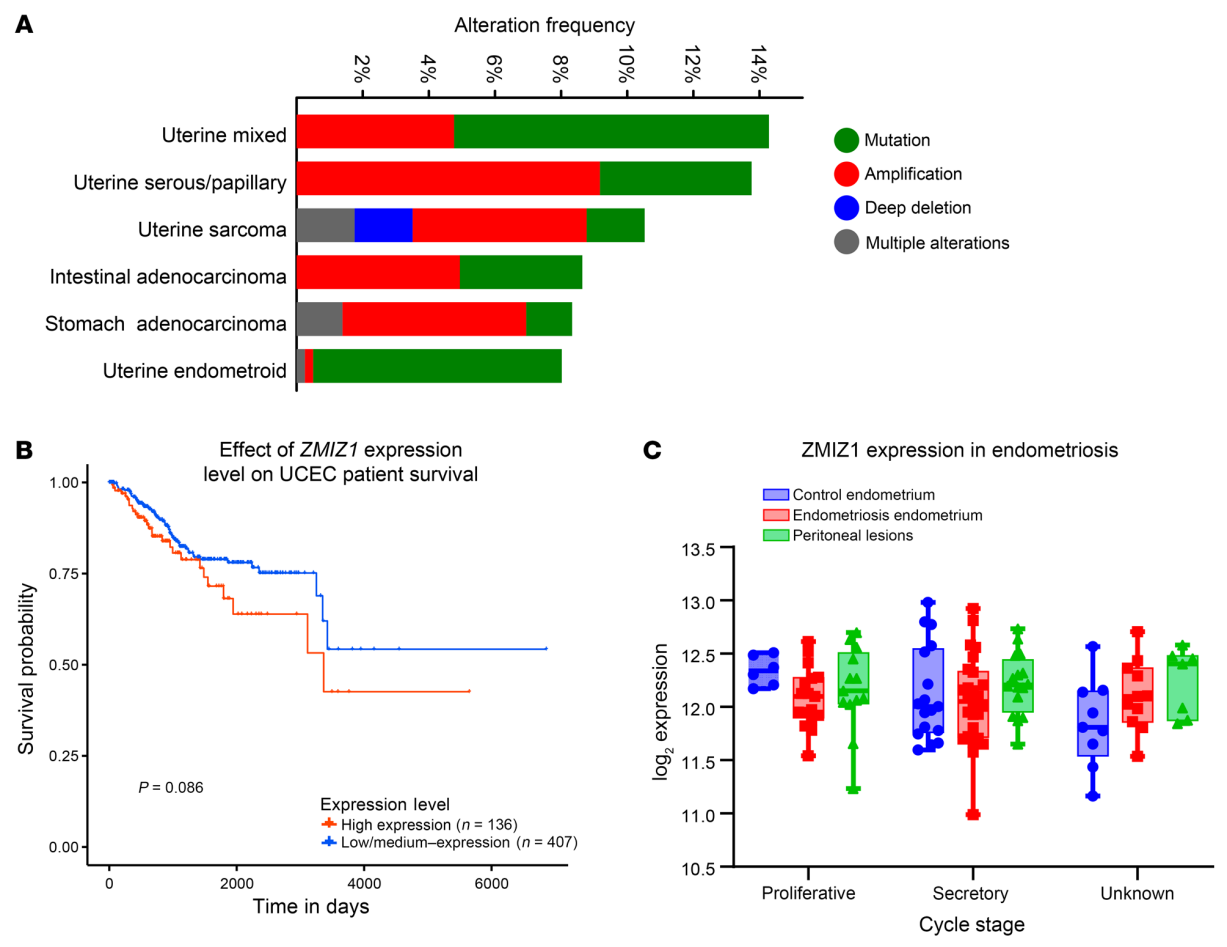
ZMIZ1 expression in endometrial pathologies. (**A**) Summary plot showing *ZMIZ1* mutation frequencies associated with tumors using cBioportal analysis of TGCA cancer dataset (31, 32). (**B**) UALCAN survival plot analysis of TGCA uterine cancer dataset based on relative level of *ZMIZ1* expression (33, 34). This shows survival probability of patients with uterine cancer in the TCGA database stratified according to level of *ZMIZ1* transcript. High expression TPM above top quartile, *n* = 136. Low/medium expression TPM below top quartile, *n* = 407. (**C**) *ZMIZ1* expression in endometrial biopsies from individuals with endometriosis compared with those without endometriosis (control) and in peritoneal endometriosis lesions using GEO GSE141549 dataset (35). Proliferative control *n* = 6 proliferative endometriosis *n* = 18, proliferative peritoneal lesions *n* = 13, secretory control *n* = 17, secretory endometriosis *n* = 26, secretory peritoneal lesion *n* = 16, unknown control *n* = 9, unknown endometriosis *n* = 11, unknown peritoneal lesion *n* = 7. Box is the mean value ± 25th to 75th percentile, whiskers show min to max. All differences are nonsignificant by unpaired 2-tailed *t* test, corrected for multiple comparisons.

*ZMIZ1* expression was detected in endometrial biopsy RNA-seq samples from both proliferative and midsecretory stages (29) with increased levels detected at the midsecretory stage (Figure 1A). Analysis of scRNA-seq in a published human endometrium dataset (30) showed that *ZMIZ1* mRNA expression was detected throughout the menstrual cycle, with lower levels at proliferative and early-to-midsecretory phases (Figure 1, B–D, and Supplemental Figure 1B). *ZMIZ1* was detected with highest expression in stromal and endothelial cell clusters. (Figure 1, B–D, and Supplemental Figure 1B). We confirmed ZMIZ1 protein expression in endometrial biopsies using IHC (Figure 2A) and observe decreased ZMIZ1 in epithelia and increased levels in stroma between proliferative and secretory phases (Figure 2A), similar to the dynamics observed in bulk and scRNA-seq analysis (Figure 1, A, C, and D). Knowing that ZMIZ1 is expressed in endometrial cells, that ZMIZ1 impacts signaling of transcription factors associated with endometrial function (TP53, NOTCH1, ESR1, SMAD), and that the *ZMIZ1* gene super enhancer interacts with ESR1, we examined whether *ZMIZ1* might be associated with diseases of the endometrium. First, using cBioportal analysis of The Cancer Genome Atlas (TGCA) data (31, 32), we found that *ZMIZ1* mutations occur in up to 14% of uterine tumors (Figure 3A). A recent study of patients with breast cancer reported that tumors with increased *ZMIZ1* expression were correlated with decreased survival of ESR1-positive breast cancer patients (20). Since uterine cancer is also estrogen sensitive, we evaluated uterine corpus endometrial cancer samples (UCEC) in TCGA for the impact of the relative level of *ZMIZ1* transcript expression on survival using the UALCAN tool (33, 34). Survival curves of patients with tumors having higher (TPM above top quartile) or lower (TPM below top quartile) expression levels were compared via log rank test (33) and exhibited a nonsignificant trend towards decreased survival in patients having increased *ZMIZ1* expression (Figure 3B). Additionally, analysis of *ZMIZ1* expression in endometrial samples in the Turku Endometriosis Bulk RNA-seq Database (35) suggests a nonsignificant decrease in *ZMIZ1* levels at the proliferative phase in patients with endometriosis compared with normal samples (Figure 3C). *ZMIZ1* transcript was expressed in peritoneal endometriosis lesions at levels comparable to the endometrium (Figure 3C). IHC analysis of samples from eutopic endometria and lesions from endometriosis patients indicated ZMIZ1 protein was detected but varied substantially between individual patients (Figure 2B). Unfortunately, cycle stage data was not available for the endometriosis samples; however, these observations indicate the importance of future investigation to characterize the impact of ZMIZ1 on endometriosis.

### ZMIZ1 regulates endometrial stroma differentiation.

**Figure 4:**
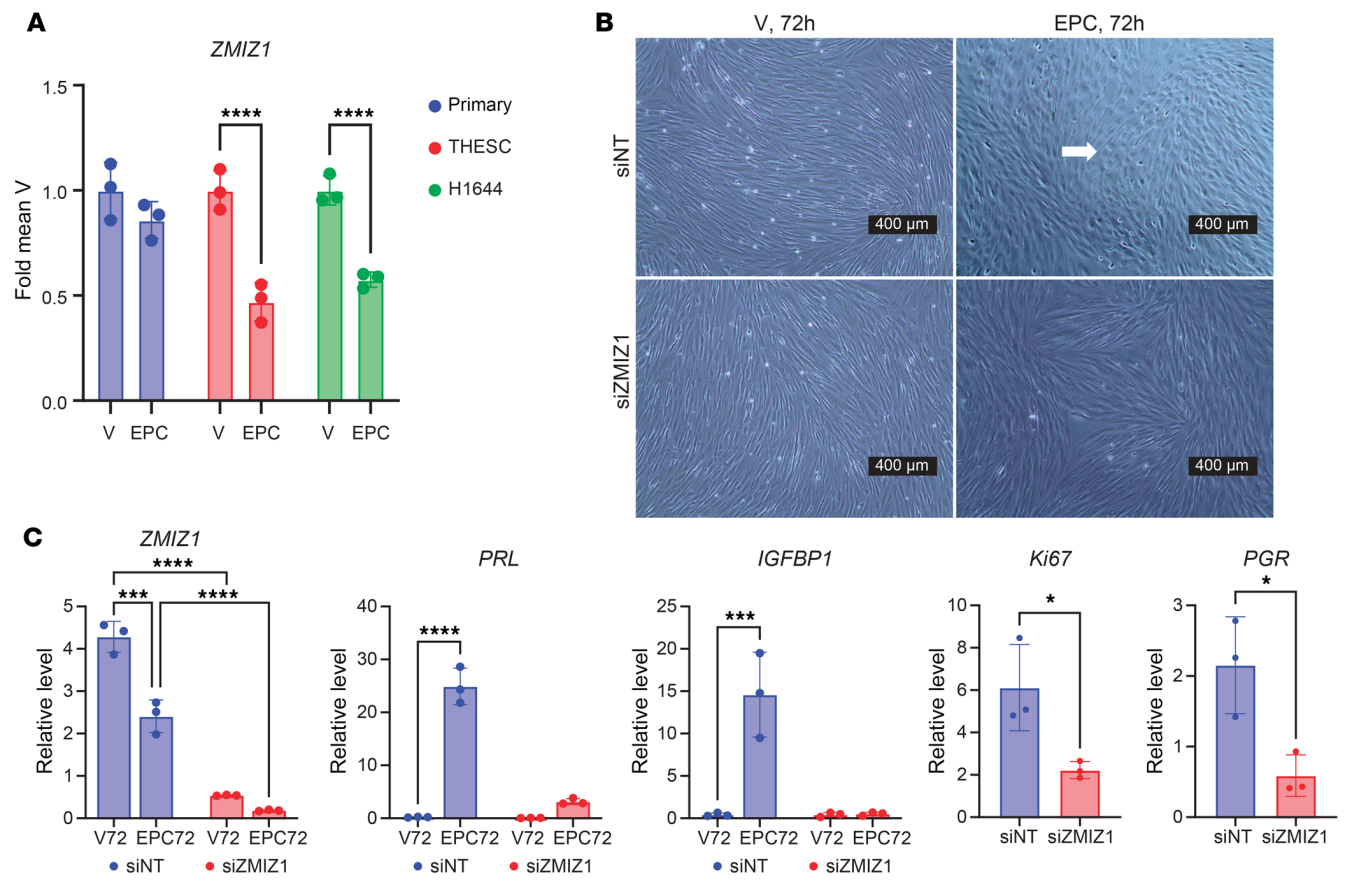
Endometrial stroma decidual response is ZMIZ1 dependent. (**A**) *ZMIZ1* expression in RNA-seq datasets from primary hESC (GSE205477; *n* = 3 each vehicle (V) or EPC treated) and from THESC or H1644 immortalized hESC (*n* = 3 each condition; *P* < 0.0001 by unpaired 2-tailed *t* test). Signal is normalized to the average vehicle of each cell line. (**B**) Phase contrast images with white arrow showing morphological change occurring following EPC-induced decidualization of H1644 cells treated with nontargeting siRNA (siNT) that does not occur following *ZMIZ1* siRNA targeting (siZMIZ). Scale bar: 400 μm. *n* = 3 each condition. (**C**) RT-PCR reveals successful knockdown of *ZMIZ1* as well as the inability of siZMIZ1-targeted cells to induce the decidual genes *PRL* and *IGFBP1*. ***P < 0.001, ****P < 0.0001, by 2-way ANOVA; *n* = 3 each condition. Decreased levels of the *Ki67* and *PGR* transcripts in nondecidualized (V treated) siZMIZ1 vs siNT-targeted cells were confirmed. *P < 0.05 by paired 2-tailed *t* test, *n* = 3.

In preparation for and during pregnancy, endometrial stromal cells undergo hormone-driven differentiation called decidualization (11, 36). To assess whether hormone-induced decidualization might regulate *ZMIZ1* expression, we analyzed RNA-seq datasets from hESC, both primary cells (37) and 2 hESC cell lines that were telomerase immortalized (THESC and H1644). *ZMIZ1* RNA was detected in all 3 cell lines (Figure 4A). Additionally, treatment with estrogen, progesterone, and cAMP (EPC) to induce decidualization significantly decreased *ZMIZ1* in the 2 immortalized hESC cell lines (Figure 4A). *ZMIZ1* was not significantly altered in decidualized primary cells; the response differences might be attributed to variations in interindividual characteristics of tissue donors used to derive the cell lines or to changes introduced by immortalization of the cells. To test the role of estrogen signaling in the decreased expression of *ZMIZ1* in THESC, estrogen was omitted (PC, Supplemental Figure 1C), which resulted in further decrease in *ZMIZ1*, suggesting that estrogen modulates the rate of *ZMIZ1* decrease during decidualization.

Because of the ESR1-binding regions at *ZMIZ1* enhancers, *ZMIZ1* expression in endometrial cells, and correlation of *ZMIZ1* modulation with endometrial disease, we hypothesized that ZMIZ1 mediates hormone-dependent uterine cell responses. The importance of *ZMIZ1* to hormone-induced decidual response of cultured human endometrial stromal cells (hESC) was tested by targeting the *ZMIZ1* transcript with siRNA (siZMIZ1). EPC-induced decidual morphological changes seen in the nontargeting siRNA control cells (siNT; Figure 4B, white arrow) fail to occur after siZMIZ1 knockdown. RT-PCR confirms that siZMIZ1 decreased *ZMIZ1* transcript (Figure 4C) and that siZMIZ1-targeted cells fail to induce the decidual genes *PRL* and *IGFBP1* (Figure 4C).

**Figure 5:**
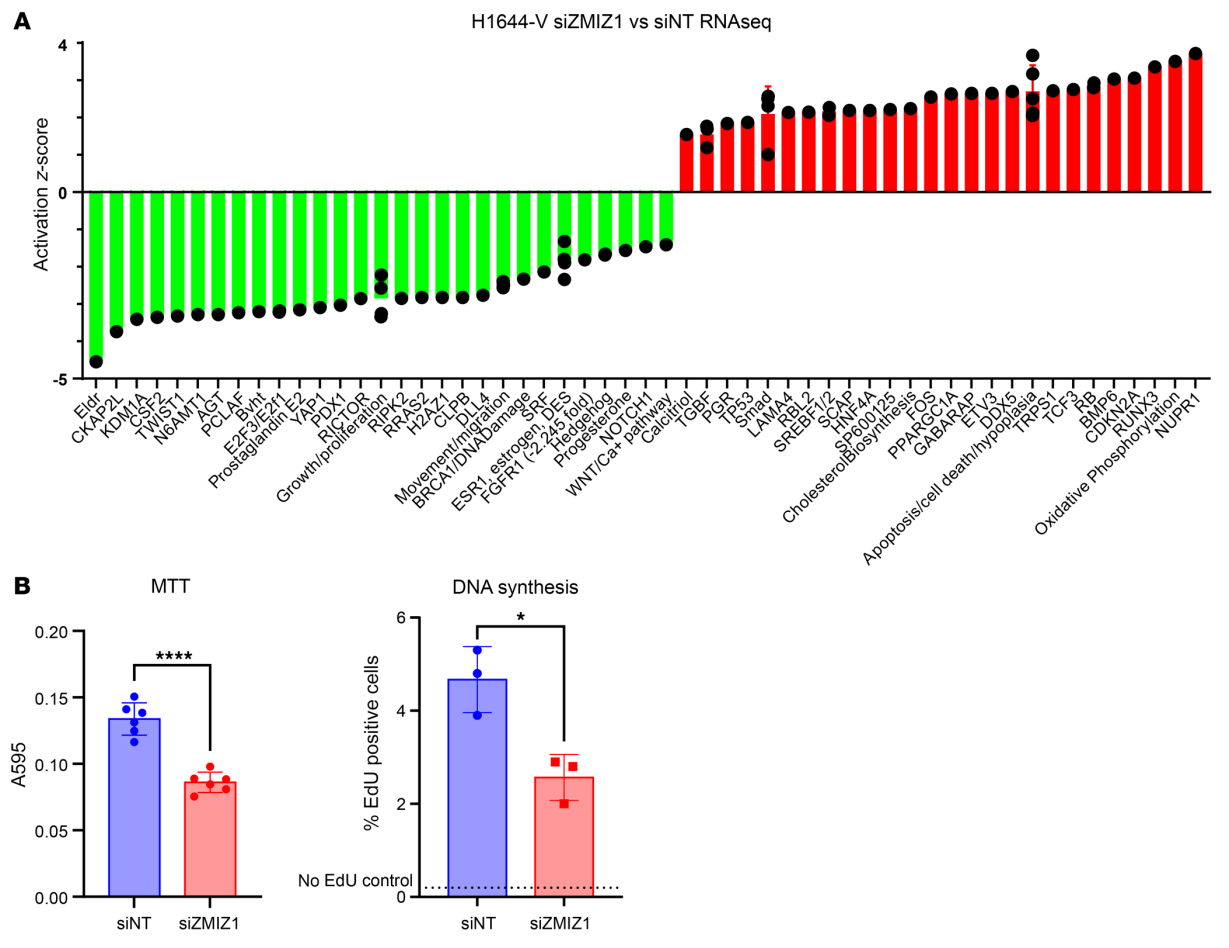
Endometrial stroma cell growth is ZMIZ1 dependent. (**A**) Graphical summary of some of the top enriched signals, functions, and pathways following IPA Core Analysis of the ZMIZ1-dependent gene set (siZMIZ1 vs siNT). Negative or positive *z* scores suggest increased or decreased activity. (**B**) Decreased cell viability (MTT assay *n* = 5; ****P < 0.0001 by unpaired *t*-test) and DNA synthesis (EdU incorporation *n* = 3 *P < 0.05 by unpaired 2-tailed *t* test) occur following ZMIZ siRNA targeting.

To understand how ZMIZ1-dependent signals might mediate hESC decidual response, we used RNA-seq to identify genes altered by *ZMIZ1* targeting and then used pathway analysis to assess perturbed cellular functions and signaling (Figure 5A and Supplemental Table 1). Since the pathway analysis suggests decreased growth/proliferation associated with siZMIZ1 versus nontargeting control siRNA differentially expressed genes (DEG), we evaluated the growth of the siNT and siZMIZ1 hESC cells. siZMIZ1-targeted cells exhibit decreased activity in a MTT assay, which measures cell viability (Figure 5B). Additionally, siZMIZ1-targeted cells exhibit decreased expression of the *Ki67* proliferative gene (Figure 4C) and decreased DNA synthesis, as reflected by EdU incorporation (Figure 5B), indicating that ZMIZ1-dependent processes impact growth of hESC cells.

### ZMIZ1 in Ishikawa endometrial cancer cell line.

To assess a potential role for ZMIZ1 in endometrial epithelial cells, we evaluated the impact of ZMIZ1 in Ishikawa cells, an epithelial cell line derived from an endometrial tumor (38). Cell viability, as reflected by an MTT assay, was not altered in siZMIZ1 versus siNT cells treated with vehicle (Supplemental Figure 1D), and was increased in siNT cells treated with estrogen, but not in estrogen-treated siZMIZ1-targeted cells, indicating that estrogen-dependent growth of Ishikawa cells is enhanced by ZMIZ1. Estrogen increased the *ZMIZ1* transcript in the siNT-targeted Ishikawa cells (Supplemental Figure 1D), and increased *PGR* and *GREB1* transcripts in both siNT and siZMIZ1-targeted cells (Supplemental Figure 1D); however, the amplitude of *PGR* increase was lessened by siZMIZ1 targeting, suggesting that ZMIZ1 plays a gene-specific role as an ESR1 coregulator.

### Zmiz1 expression in the mouse.

**Figure 6:**
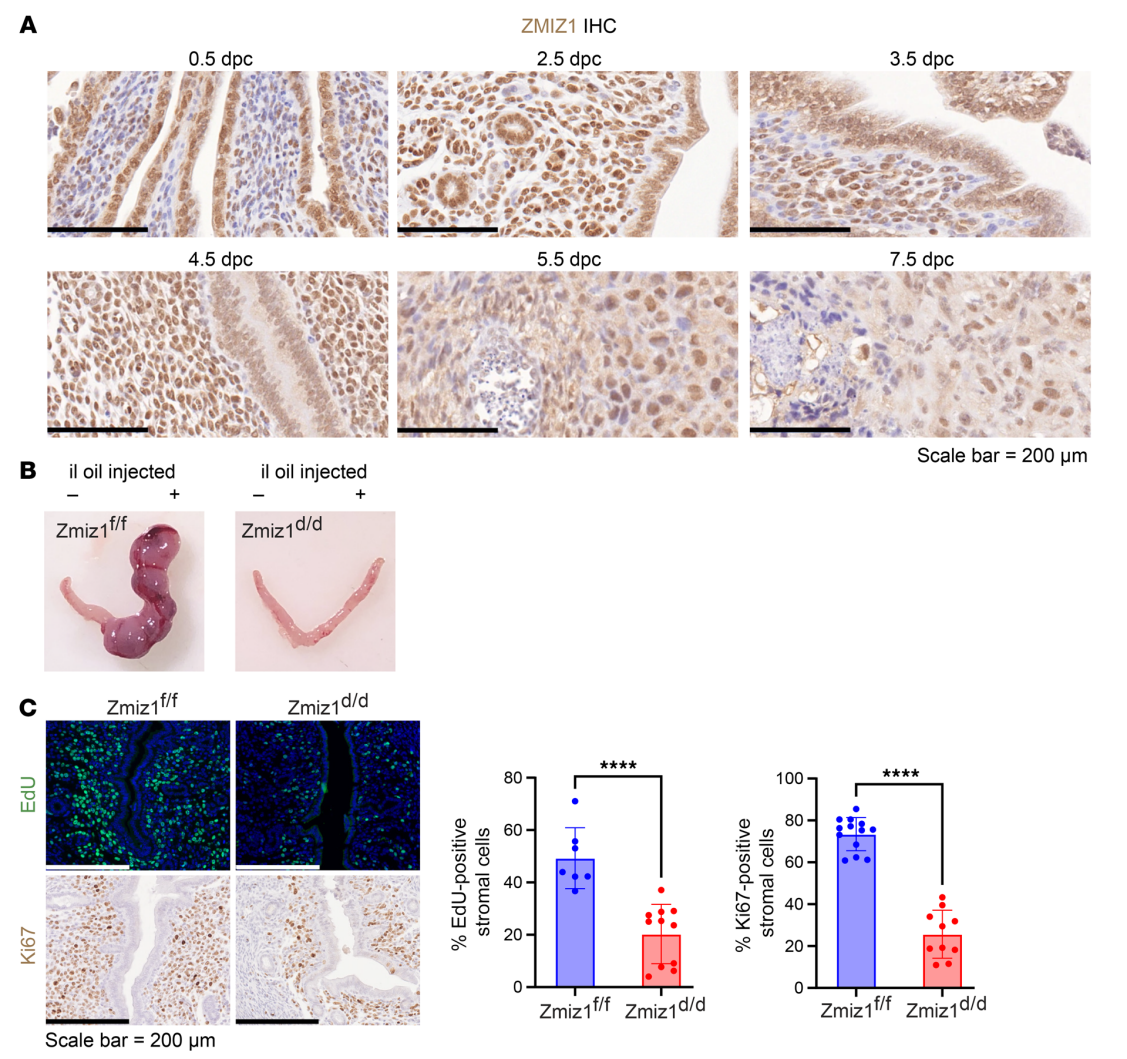
ZMIZ1 modulates proliferative-decidual switching of mouse uterine stromal cells. (**A**) ZMIZ1 IHC of mouse uterine samples from early pregnancy collected on the morning after mating 0.5 dpc or on 2.5, 3.5, 4.5, 5.5, and 7.5 dpc. Scale bar = 200 μm. (**B**) Decidual response of Zmiz1^d/d^ and Zmiz1^f/f^. Note increased size of the Zmiz1^f/f^ uterine horn injected intraluminally with oil (il oil +) compared with the uninjected side (–). (**C**) Analysis of stromal cell proliferation 20 hours after il oil injection of Zmiz1 ^f/f^ and Zmiz1^d/d^ mice by EdU incorporation or Ki67 IHC. Percent EdU or Ki67-positive stromal cells were counted in Image J. For EdU samples *n* = 10 Zmiz1^f/f^ and 11 Zmiz1^d/d^; for Ki67, *n* = 13 Zmiz1^f/f^ and 10 Zmiz1^d/d^; ****P < 0.0001 by unpaired 2-tailed *t* test.

The role of *Zmiz1* in regulating uterine biology in vivo was investigated using mouse models. First, we characterized mouse ZMIZ1 expression in early pregnancy using IHC analysis. ZMIZ1 is detected in epithelial cells prior to implantation, beginning on 0.5 days postcoitus (dpc) (Figure 6A), with increasing stroma cell expression later (2.5–3.5 dpc; Figure 6A). Following embryo implantation, epithelial expression decreases, and stromal expression is maintained (4.5 dpc; Figure 6A). ZMIZ1 is detected in decidualized stromal cells as well (5.5–7.5 dpc; Figure 6A). The uterine ZMIZ1 expression pattern during early pregnancy is similar to that of PGR, an ESR1 regulated gene (39, 40).

### Zmiz1 ablation results in infertility.

Since *Zmiz1*-null mice exhibit embryonic lethality, conditional ablation of *Zmiz1* in the reproductive axis was conducted (41). Mice with a conditional allele for *Zmiz1* (Zmiz1^f/f^) (21) were crossed to the Pgr^Cre^ (42) to generate the Zmiz1^d/d^ mouse. This Zmiz1^d/d^ mouse will exhibit ablation of *Zmiz1* in all *Pgr-*expressing cells of the uterus, pituitary, and ovary. Expression of ZMIZ1 protein and transcript was decreased in the uteri of Zmiz1^d/d^ mice compared with uteri from littermate Zmiz1^f/f^ mice (Supplemental Figure 2, A–C). Western blot revealed that the full-length ZMIZ1 protein is expressed in Zmiz1^f/f^ uterine tissue (Supplemental Figure 2B). The fertility of Zmiz1^d/d^ females was evaluated by continuous breeding; no pups were born to 6 Zmiz1^d/d^ females during an interval in which 6 Zmiz1^f/f^ females delivered 29 litters with an average of 6.6 pups/litter (Supplemental Figure 3A). At the end of the breeding trial, Zmiz1^d/d^ uterine tissue exhibited abnormal gland pathology and extensive fibrosis (not shown) that was also seen in a cohort of Zmiz1^d/d^ females aged to 27–30 weeks (Supplemental Figure 3B). Atypical features in aged Zmiz1^d/d^ uteri included pleomorphic disorganized glandular epithelium with amorphous eosinophilic material surrounding glands in gland clusters. No hyperplasia or neoplasia were seen. These gland abnormalities occurred in 6 of 8 of the aged Zmiz1^d/d^ but were not seen in any of the 6 age-matched Zmiz1^f/f^ samples or in samples from younger mice. Uterine collagen deposition occurs with aging (43) but is accelerated in Zmiz1^d/d^ tissue (Supplemental Figure 3B) and was observed in 2 of 6 Zmiz1^f/f^ and in 8 of 8 Zmiz1^d/d^. The collagen deposition was limited to the endometrium, not found in the myometrium, nor were there any fibroids observed (Supplemental Figure 3B).

Estrous cyclicity (Supplemental Figure 3C), embryo transport to the uterus on 3.5 dpc, and ovarian histology of Zmiz1^d/d^ and Zmiz1^f/f^ were comparable (Supplemental Figure 3D). Although Zmiz1^d/d^ ovulated in response to exogenous gonadotrophins, fewer oocytes were released, and although serum prolactin levels were comparable, serum progesterone levels of 3.5 dpc Zmiz1^d/d^ were reduced (Supplemental Figure 3D), indicating that ovarian dysfunction contributed to the infertility. The infertility may have a contribution from *Zmiz1* deletion in the pituitary and ovary, resulting in disruption on the neuroendocrine control of pregnancy in the mouse.

### Zmiz1 deletion impairs decidualization in mice.

**Figure 7:**
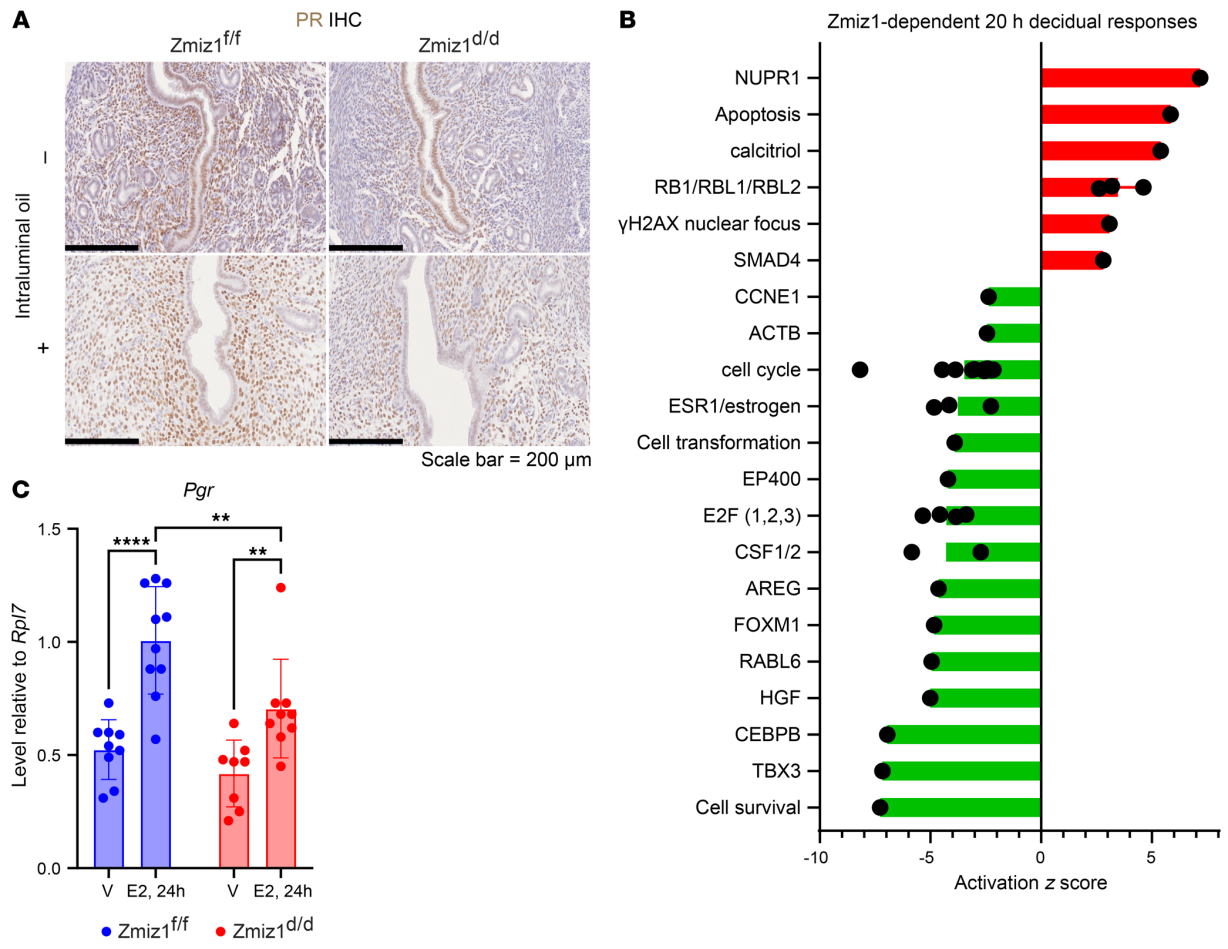
ZMIZ1 Modulates progesterone receptor expression of mouse uterine cells. (**A**) PGR IHC of Zmiz1^f/f^ and Zmiz1^d/d^ tissue from an uninjected (–) uterine horn, or 20 hours after intraluminal (il) oil injection (+) reveals decreased stromal PGR in Zmiz1^d/d^. Scale bar: 200 μm. *n* = 7 Zmiz1^f/f^ (–); *n* = 7 Zmiz1^f/f^ (+); *n* = 5 Zmiz1^d/d^ (–); *n* = 6 Zmiz1^d/d^ (+). (**B**) Graphical summary of some of the top enriched signals, functions, and pathways following IPA Core Analysis of DEG from microarray analysis of Zmiz1^d/d^ vs Zmiz1^f/f^ RNA 20 hours after il oil injection. Positive or negative *z* score suggests *Zmiz1* deletion increases or decreases signaling, respectively. (**C**) RT-PCR for *Pgr* of uterine RNA from Zmiz1^f/f^ or Zmiz1^d/d^ ovariectomized females treated with vehicle (V) or estradiol (E2) for 24 hours. *n* = 10 Zmiz1^f/f^ V and E2; *n* = 8 Zmiz1^d/d^ V *n* = 9 Zmiz1^d/d^ E2. **P < 0.01, ****P < 0.0001 by 2-way ANOVA.

Because the reduced serum progesterone (Supplemental Figure 3D) would confound evaluation of uterine responses, ovariectomy and exogenous hormone treatment was used to evaluate decidualization of Zmiz1^d/d^ versus Zmiz1^f/f^ female mice. Decidual response was *Zmiz1* dependent, with no uterine weight increase, morphological changes, or induction of expression of decidual transcripts of *Bmp2* and *Prl8a2*. (Figure 6B and Supplemental Figure 4, A–C). Although decidualization decreased *ZMIZ1* transcript in hESC (Figure 4, A and C), in the mouse uterus, decidualization did not change the level of *Zmiz1* transcript (Supplemental Figure 4A). This indicates that, although the function of ZMIZ1 is conserved between species, its regulation differs. Stromal cell proliferation 20 hours after intraluminal (il) oil injection (decidual stimulus) was significantly reduced in Zmiz1^d/d^ compared with Zmiz1^f/f^ as reflected by EdU incorporation or Ki67 expression (Figure 6C). Decidual response requires dynamic changes in PGR, with decreased epithelial and increased stromal levels, to mediate proliferative/decidual switching (PDS) of uterine cells (44). In Zmiz1^f/f^ uterine tissue, PGR decreased in epithelial cells and increased in stromal cells after the decidual stimulus (Figure 7A), whereas stromal cell PGR did not increase in Zmiz1^d/d^ samples (Figure 7A). Western blot analysis revealed that PGR-B isoform level was lower in Zmiz1^d/d^ uterine protein extracts (Supplemental Figure 4D).

### Zmiz1 impacts the uterine transcriptome.

To assess how uterine deletion of *Zmiz1* impacts uterine hormonal responses, transcriptomic microarray analysis was used to compare uterine RNA in the unstimulated or stimulated uterine horn of mice undergoing a hormonal induced decidual response. Comparing RNA isolated from the unstimulated horns of Zmiz1^d/d^ versus Zmiz1^f/f^ mice, differential gene expression (DEG) was minimally impacted by *Zmiz1* deletion (2-fold, *P* < 0.05, 577 DEG; Supplemental Figure 5A). However, analysis of the stimulated horn DEG indicated that 1,381 genes were differentially expressed after *Zmiz1* deletion (Supplemental Figure 5A). Ingenuity Pathway Analysis (IPA) was used to find enriched functions and signaling pathways in the Zmiz1-dependent gene signature (Supplemental Table 2, Figure 7B, and Supplemental Figure 5B). Consistent with the decreased stromal proliferation observed (Figure 6C), the transcriptomic analysis indicated decreased cell cycle progression (Supplemental Table 2 and Figure 7B). The induction of cell cycle progression genes is inhibited in the absence of *Zmiz1* (Supplemental Figure 5, B and C), suggesting that Zmiz1 works to enhance gene responses necessary for decidual transformation.

Since PGR is increased via ESR1-dependent induction (11), and PGR expression was decreased in Zmiz1^d/d^ uterine stromal cells (Figure 7A), ESR1 was evaluated to determine if change in ESR1 expression was the cause of the decrease in PGR. Western blot for ESR1 indicated a trend toward an increase in expression in samples from Zmiz1^d/d^ uteri that did not undergo decidual stimulus (Supplemental Figure 4D), with comparable levels in stromal cells (Supplemental Figure 4D), indicating ESR1 expression was not impacted in a way that might explain the decreased stromal cell PGR induction. Estrogen regulation of *Pgr* transcript was examined in ovariectomized mice given a single estrogen injection. *Pgr* was increased by estrogen in both Zmiz1^f/f^ and Zmiz1^d/d^ samples (Figure 7C), however the amplitude of the response was *Zmiz1* dependent, suggesting interaction between ZMIZ1 and ESR1 mediated responses.

### Zmiz1 impacts estrogen-induced gene expression.

**Figure 8:**
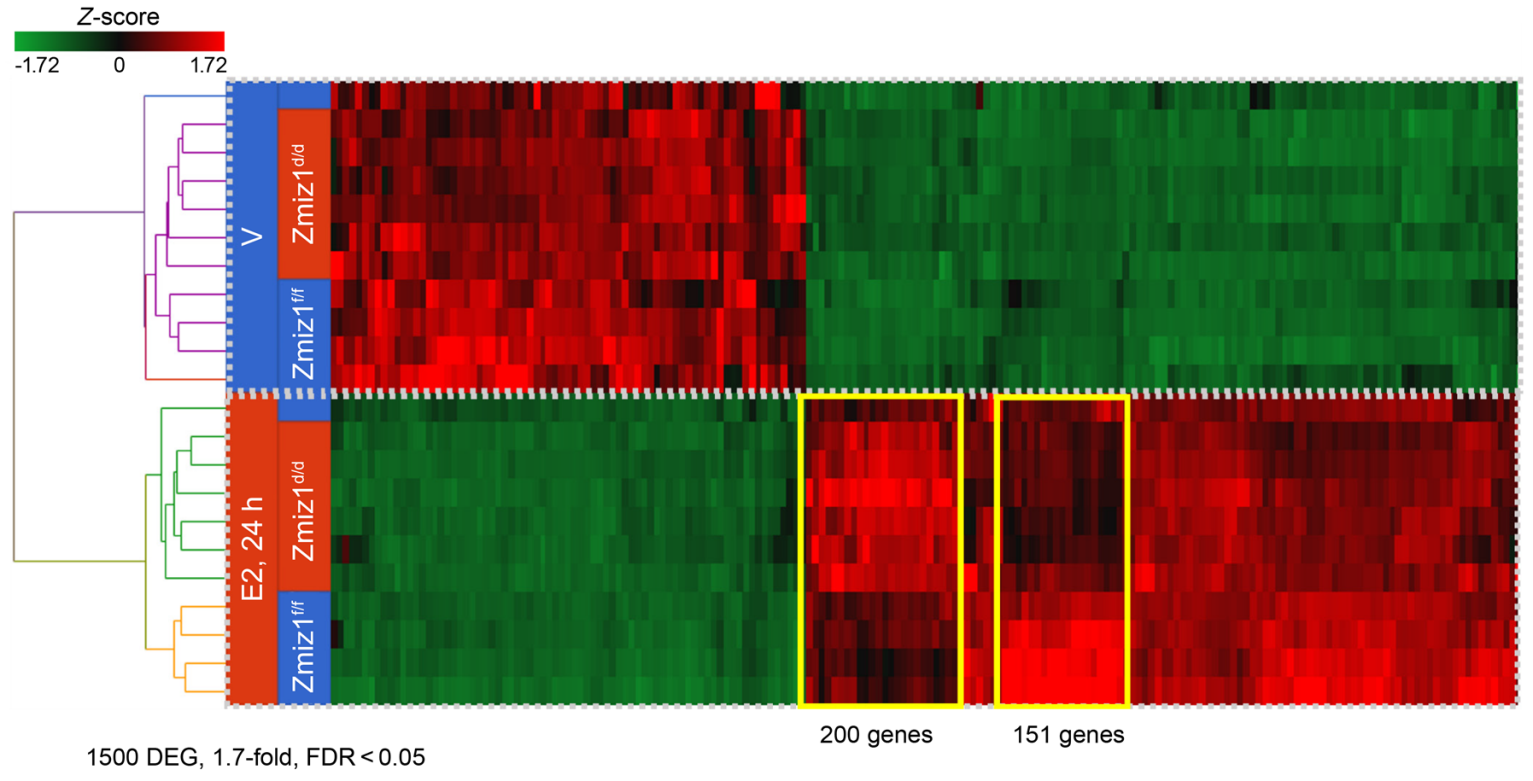
ZMIZ1 regulates uterine estrogen response. Hierarchical clustered heatmap of 1500 DEG in Zmiz1^f/f^ and Zmiz1^d/d^ uterine RNA from vehicle (V) or E2 treated mice. Sample and gene orders are the result of the clustering. Clusters where E2 response of Zmiz1^d/d^ is greater than Zmiz1^f/f^ (200 genes) or E2 response of Zmiz1^d/d^ is less than Zmiz1^f/f^ (151 genes) are outlined with yellow boxes.

To assess whether uterine deletion of *Zmiz1* globally impacts estrogen responses, RNA from ovariectomized females treated with vehicle or estrogen was compared using RNA-seq. The basal levels (Vehicle treated) of 1,500 estrogen-regulated DEG (Figure 8; 1.7-fold, FDR < 0.05) were minimally impacted by *Zmiz1* deletion. Two prominent clusters of estrogen-regulated genes altered by *Zmiz1* deletion were observed: a 200-gene cluster with higher fold increase in Zmiz1^d/d^ than Zmiz1^f/f^ and a 151 gene cluster with increased induction in Zmiz1^f/f^ (Figure 8). Pathway analysis of the 200 gene cluster exhibiting increased estrogen response in *Zmiz1*-deleted mice included enrichment of collagen biosynthesis and fibrosis (Table 1 and Supplemental Table 3). This finding is interesting in light of the accelerated uterine fibrosis observed in Zmiz1^d/d^ mice with increasing age (Supplemental Figure 3B). The Zmiz1^d/d^ exhibit normal estrous cycles (Supplemental Figure 3C), which includes a preovulatory rise in circulating estrogen with each cycle (45) and suggests that the fibrosis is driven by accumulated lifetime estrogen signaling. RT-PCR analysis confirms increased estrogen response of several collagen subunit transcripts (Supplemental Figure 6A). However, no fibrosis or increased collagen deposition (as indicated by Masson’s Trichrome stain) is observed in Zmiz1^d/d^ uterine sections (Supplemental Figure 6B) following a single (24 hours) estrogen treatment of mice that were ovariectomized prior to the onset of the fibrosis phenotype (Supplemental Figure 3B).

**Figure 9:**
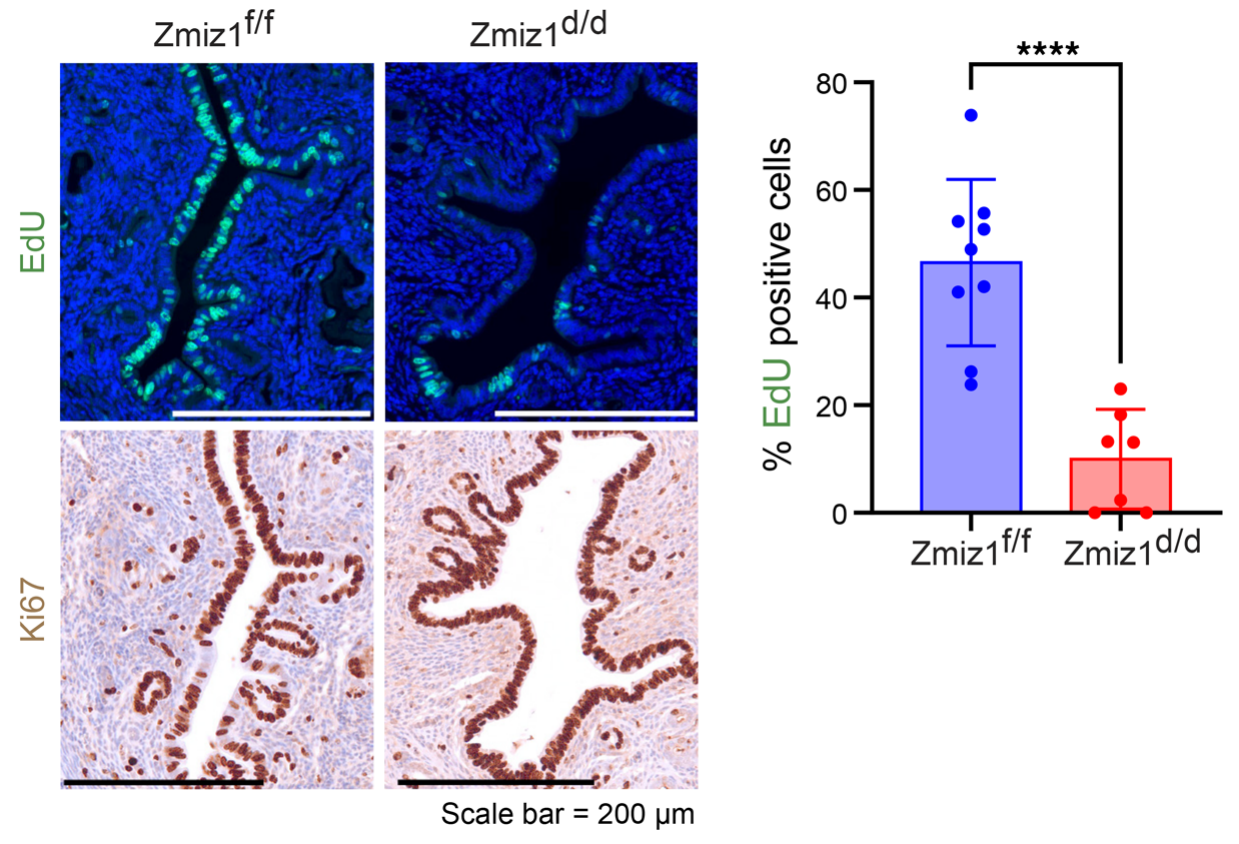
ZMIZ1 regulates uterine estrogen-dependent epithelial cell proliferation. EdU incorporation and Ki67 IHC of Zmiz1^f/f^ and Zmiz1^d/d^ uterine tissue treated for 24 hours with E2. Scale bar: 200 μm. Percent EdU positive cells were counted in Image J. *n* = 9 Zmiz1^f/f^ and 7 Zmiz1^d/d^. ****P < 0.0001 by unpaired 2-tailed *t* test. *n* = 6 each sample.

Pathway analysis of the 151-gene cluster with Zmiz1-dependent estrogen response included enrichment of multiple cell cycle progression pathways (Table 2 and Supplemental Table 4). As was seen in the decidual Zmiz1-dependent gene profiles, the amplitude of estrogen-dependent cell cycle progression genes is decreased (Supplemental Figure 6C). These findings suggest a role for ZMIZ1 in optimizing the estrogen-responsive gene expression during estrogen-induced uterine epithelial cell proliferation (12). Epithelial cell DNA synthesis, as reflected by EdU incorporation, was inhibited by *Zmiz1* deletion (Figure 9), however the Zmiz1^d/d^ epithelial cells were positive for the broad cell-cycle proliferative marker Ki67 (Figure 9), suggesting that *Zmiz1* is needed for progression through S-phase. Further IPA analysis focusing on *Zmiz1*-dependent differences in estrogen-treated samples obtained by comparing differences between the estrogen-treated samples (E2, 24h Zmiz^d/d^ vs E2, 24h Zmiz1^f/f^; 149 DEG) also emphasized that fibrosis- and proliferation-associated functions were increased or decreased, respectively, by loss of *Zmiz1* (Supplemental Figure 6D and Supplemental Table 5).

The magnitude of uterine growth and underlying gene responses are reduced when *Zmiz1* is disrupted. Similarly, targeting *ZMIZ1* decreased breast cancer cell growth, due to decreased E2F2 induction via interaction between ZMIZ1 and ESR1 (20). As was seen in the hESC following siRNA targeting of ZMIZ1 (Figure 5A and Supplemental Table 1), pathway analyses indicate that targeting mouse uterine *Zmiz1* inhibits estrogen response (Figure 7B, Supplemental Figure 6D, and Supplemental Tables 2 and 5). Therefore, potential interaction between ESR1 and ZMIZ1 in uterine tissue was examined by immunofluorescent colocalization. In tissue from WT mice treated with exogenous hormones, without a decidual stimulus (Supplemental Figure 7A), as well as in tissue from mice treated with estrogen (Supplemental Figure 7B), both ZMIZ1 and ESR1 were detected in nuclei of luminal and glandular epithelial cells and stromal cells (Supplemental Figures 7, A and B), with especially prominent expression in glandular epithelial cells.

## Discussion

Uterine health is impacted by endogenous estrogen signaling as well as by exposure to environmental endocrine disruptors (46). Understanding the mechanisms by which estrogen signals through the ESR1, and identifying the factors that modify ESR1 action, are critical to elucidating how uterine biology is impacted by estrogen. Epigenomic enhancer analysis identified *Zmiz1* colocation at the highest ranked mouse uterine ESR1-binding super enhancer (14); a similar enhancer landscape is observed at the human *ZMIZ1* gene (Supplemental Figure 1A). Expression of ZMIZ1 RNA and protein in multiple uterine cell types and the analysis of clinical datasets showing *ZMIZ1* mutations in endometrial cancer and the potential impact of *ZMIZ1* expression levels on endometrial cancer and endometriosis underlines its possible importance in uterine function (Figures 1–3). Indeed, a previous study identified *ZMIZ1* as one of multiple genes upregulated in ectopic versus eutopic endometrium of women with endometriosis (47). Here, we defined the role of ZMIZ1 in the mouse and human uterus and show that it is critical for endometrial stromal cell differentiation and estrogen signaling.

Knocking down the levels of *ZMIZ1* in hESC using siRNA demonstrated that it was critical for stroma cell decidualization and for regulating cell proliferation and PGR expression. (Figures 4 and 5). Paradoxically, *ZMIZ1* mRNA is decreased in decidualized hESC cells (Figure 4, A and C), suggesting it is needed for the differentiation driving decidualization but is not needed once the cells have differentiated.

Using a mouse as an in vivo model, we also demonstrated that *Zmiz1* was critical for uterine stromal cell decidualization, endometrial cell proliferation, and the regulation of stromal cell PGR expression. (Figures 6, 7, and 9, and Supplemental Figure 4). These impairments likely underlie inability to achieve decidual phenotype, as *Pgr* deletion also prevents decidualization (11, 48), and progesterone-driven epithelial cell differentiation, together with sufficient stromal cell expansion, is needed to support implanting embryos; this process has been dubbed proliferation-differentiation switching (49, 50). We observed that, in human endometrium scRNA-seq, *ZMIZ1* transcripts were expressed at lower levels in epithelial cells than in stromal cells (Figure 1, B–D), and we do observe clear impact on stromal responses; however, we did also observe an impact of targeting *ZMIZ1* on estrogen responses of growth and induction of *PGR* transcript expression in Ishikawa cells (Supplemental Figure 1D), indicating its importance in both epithelial and stromal cell types.

The trend towards decreased expression of *ZMIZ1* transcript in endometriosis versus normal eutopic endometrium, together with our observation that targeting uterine ZMIZ1 impairs PGR expression, suggests that ZMIZ1 may impact development of endometriosis by contributing to progesterone resistance, and future work to examine this possible mechanism might yield endometriosis therapies that target ZMIZ1.

ZMIZ1’s importance in cell growth has been observed in multiple tissues, including decreased growth of MEFs isolated from ZMIZ1-null embryos (41) or siZMIZ1-targeted human dermal lymphatic endothelial cells (17) as well as decreased estrogen-dependent proliferation of siZMIZ1-targeted breast cancer cells (20). *ZMIZ1* disruption inhibited estrogen-dependent growth of Ishikawa cells, which are derived from endometrial cancer, suggesting development of ZMIZ1-targeting might be therapeutic. In the breast cancer cell study, interaction between ESR1 and ZMIZ1 to regulate cell cycle progression genes was hypothesized, with demonstrated interactions at the E2F2 gene promoter (20). RNA-seq analysis of siZMIZ1 versus siNT-targeted hESC cells or of estrogen-treated or decidualized Zmiz^d/d^ vs Zmiz1^f/f^ mouse uterine tissue indicates decreased E2F signaling (Figure 5A, Figure 7B, Supplemental Figure 6D, and Supplemental Tables 1, 2, and 5), suggesting ZMIZ1’s potential role via E2F signaling in uterine cells as well. In the mouse uterine tissue, ZMIZ1-dependent E2F targets include *Ccna2*, which recently was demonstrated to have crucial roles in maintaining pregnancy (51). Evidence that CCNA2 is involved in hormone-dependent uterine activity includes in vitro observations that the CCNA2/CDK2 complex modulates ESR1 and PGR responses directly (52, 53), and functions to initiate S phase cell cycle progression (54). Our observation of decreased decidual or estrogen induction of *Ccna2* expression after *Zmiz1* deletion (Supplemental Figure 5C and Supplemental Figure 6C) suggests that ZMIZ1 impacts hormone-dependent cell cycle progression in mouse uterine cells via E2F-mediated CCNA2 regulation. CCNA2’s role in cell cycle progression includes CCNA2/CDK2 activation of FOXM1, which promotes progression from G1-S to G2 (55). FOXM1 is needed for cell cycle progression of decidualizing uterine stromal cells (56, 57) and is expressed at lower levels in siZMIZ1-targeted hESC (Supplemental Table 1) and in decidualizing Zmiz1^d/d^ versus Zmiz1^f/f^ uterine tissue (Supplemental Figure 5C). Additionally, in estrogen-treated samples, *Zmiz1* deletion results in a gene signature consistent with inhibited FOXM1 signaling (Supplemental Table 5 and Supplemental Figure 6, C and D). Altogether, these observations suggest that ZMIZ1 engagement with mediators of hormone-induced uterine cell proliferation facilitates optimal growth responses underlying uterine biology.

ZMIZ1 modulates TGFβ signaling via its impact on SMAD proteins (15, 19). In the uterus, TGFβ signaling serves to curb the extent of estrogen-dependent epithelial proliferation, which is illustrated by the epithelial overgrowth of Smad2/3 targeted uterine tissue (58, 59) and the growth promoting effect of the TGFβ inhibitor A-083-01 on uterine epithelial cells cultured as organoids (60). In siZMIZ1-targeted hESC cells and Zmiz1^d/d^ uterus, transcriptional changes suggest enhanced SMAD/TGFβ signaling (Figure 5A, Figure 7B, and Supplemental Figure 6D). In the context of estrogen stimulation, increased TGFβ activity would inhibit epithelial cell proliferation, thus, likely contributing to the decreased DNA synthesis observed (Figure 9). Additionally, increased SMAD/TGFβ signaling contributes to fibrosis in various tissues (61), including development of uterine fibroids (62). We did not observe any fibroids in Zmiz1^d/d^ uterine samples, and the accelerated collagen deposition we observed was limited to the endometrium. In any case, increased TGFβ signaling following *Zmiz1* deletion may contribute to accelerated age-related fibrosis we observe (Supplemental Figure 3B).

Transcriptomic analysis of human endometrial stroma cells, decidualized mouse uteri or mouse uteri challenged with estrogen show that loss of ZMIZ1 has little impact on the unstimulated uterine cells. The major impact of ZMIZ1 ablation on endometrial gene expression occurs when the cells or tissue are given a hormonal treatment. This highlights the role of ZMIZ1 as a coregulator of liganded ESR1 signaling. We have demonstrated that ZMIZ1 and ESR1 show colocalization in mouse endometrial stroma and epithelial cells (Supplemental Figure 7). However, we do not have direct evidence of protein-protein interactions between ZMIZ1 and ESR1. Work using breast cancer derived cell lines has shown interaction between ZMIZ1 and ESR1 by rapid immunoprecipitation mass spectrometry of endogenous proteins (RIME) and cellular colocalization (20), and it seems likely that ZMIZ1 is also interacting with ESR1 in mouse uterine cells to modulate gene responses.

ZMIZ1 has a broader role in reproduction beyond that in uterine biology. Using the Pgr^Cre^ model results in Cre-recombinase activity in the pituitary, in preovulatory granulosa cells, and in the uterus. The ability of the mice to undergo a normal estrus cycle and ovulate shows that ZMIZ1 has a minimal effect on the pituitary ovarian axis. However, the mice showed reduced ovulation and reduced levels of serum progesterone while maintaining normal prolactin levels at pregnancy day 3.5. This indicates that PgrCre-mediated *Zmiz1* deletion did not impact regulation of pituitary prolactin production or secretion but did decrease the ability of the corpus luteum to produce progesterone. This highlights the importance of this coregulator in regulating the reproductive axis. Future work to characterize relative contributions of ovarian and uterine ZMIZ1 will help clarify specific roles of these components of the reproductive system.

Because of the decreased ovarian function, we utilized ovariectomy and exogenous hormone treatments to characterize the impact of ZMIZ1 on uterine function. Our studies clearly demonstrate important roles for ZMIZ1 in endometrial function. ZMIZ1’s uterine activity modulates the amplitude of estrogen responses, impacting stromal PGR expression, perturbing proliferative/decidual switching, thereby attenuating both epithelial and stromal proliferation, precluding decidualization of stromal cells and accelerating age-related fibrosis. These ZMIZ1 activities could initiate endometrial pathologies, including uterine cancer, endometriosis, or fibroids. Future work will further characterize molecular details driving ZMIZ1-dependent signals in endometrial cells. Ours is the first study that demonstrates the importance of ZMIZ1 as an ESR1 coregulator in uterine biology and pathology.

## Methods

### Sex as a biological variable.

Our study focused on the female reproductive tract; therefore sex was not considered as a biological variable.

### Analysis of published single-cell RNA-seq data.

A publicly available published cycling human endometrium 10x Chromium scRNA-seq dataset was downloaded from Gene Expression Omnibus (GSE111976) (30) and analyzed in the R programming language. The gene-by-cell matrix was loaded from the “GSE111976_ct_endo_10x.rds” file and analyzed using Seurat (63) v. 5.1.0. Metadata, including cell ID, donor, cell type, and phase from the original publication were added using the function “AddMetaData.” UMAP coordinates from the original publication (GSE111976_umap_endo_10x.csv”) were applied to the Seurat object, and the “SCTransform” function was used to normalize gene expression for plotting. A dot plot displaying the relative expression of *ZMIZ1* by cell type and cycle phase was generated by extracting the percentage of positive cells and average expression by group using dplyer (64) v. 1.1.4 and plotting with ComplexHeatmap (65) v. 2.18.0 based on previously published code (66). The bar graph was created using ggplot2 v. 3.5.1 (67)

### Cell lines.

Immortalized human endometrial stromal cell line H1644 was derived under a protocol approved by the Institutional Review Boards of Michigan State University and Spectrum Health Medical System. Prior to participation, written informed consent was obtained from the patient. Normal human endometrial stromal cells (hESCs) were isolated from a secretory endometrial biopsy collected from a 38-year-old white woman who had been diagnosed with a benign cyst in the fallopian tube. These hESCs were cultured using hESC media (Dulbecco’s modified Eagle’s medium (DMEM)/F-12 (Gibco), supplemented with 10% charcoal-dextran treated FBS (CDS-FBS; Gibco), 1X Penicillin/Streptomycin (Gibco), and 1X sodium pyruvate (Gibco). The hESCs were subsequently infected twice with the hTERT lentiviral vector supernatant in the presence of 8 μg/ml polybrene, following the protocol as previously described (68). The hTERT-infected cells were subjected to selection in fresh medium supplemented with 25 μg/ml hygromycin. Following an 8-day selection period, the immortalized ESCs (iESCs) were maintained in growth medium with 10 μg/ml hygromycin for expansion. The iESCs were confirmed to be negative for mycoplasma contamination and exhibited an apparently normal female karyotype. Immortalized human endometrial stromal cell lines (hESC) THESC and H1644 was cultured as described (68, 69).

Ishikawa cells were purchased from Sigma and grown in Ishikawa media (phenol-free RPMI media (Gibco) supplemented with 10% charcoal-dextran–treated FBS (CDS-FBS; Gibco) and 1X Penicillin/Streptomycin (Gibco).

### siRNA targeting.

100,000 H1644 cells per well or 500,000 Ishikawa cells per well were seeded in 6-well dishes and cultured in hESC media (H1644 cells) or in Ishikawa media (Ishikawa cells) for 24 hours. Wells were washed with PBS and then cultured with 1 ml of transfection media (OptiMEM, Gibco Thermo Fisher) with 2% Charcoal Stripped Serum (Gibco Thermo Fisher). For each well, 3 μl (H1644 cells) or 5 μl (Ishikawa cells) of 20 μM siNT (control) or 20 μM siZMIZ1 siRNA (ON-TARGETplus siRNA Smartpool, Dharmacon/Horizon Discovery) was diluted to 100 μl in OpitMEM and 5 μl of RNAiMax (Thermo Fisher) was diluted to 150 μl with OptiMEM and pre incubated for 10 minutes. siRNA and RNAiMax mixtures were combined, incubated for 20 minutes, and added to cells. Cells were incubated for 48 hours prior to induction of decidual response or plating for MTT assay or DNA synthesis assay.

### Decidual response.

Media was changed to decidual media (OptiMEM with 2% Charcoal Stripped Serum and 1% Penicillin-Streptomycin) containing either 0.2% Ethanol (vehicle) or 10 nM estradiol (Sigma Chemical), 1 μM medroxyprogesterone acetate (Sigma Chemical), and 100 μM dibutyral cyclic AMP (Sigma Chemical; EPC). Media was changed after 2 days and cells were imaged (EVOS microscope, Thermo Fisher) and collected on the third day of decidualization for RNA isolation (RNA-seq and RT-PCR), for ChIPseq or HiC.

### H3K27Ac ChIPseq.

H1644 cells were fixed and isolated using Active Motif’s ChIP Cell Standard Sample Preparation Protocol and sent to Active Motif Inc. for HistonePath library preparation. Resulting libraries were submitted to the NIEHS Epigenomics and DNA Sequencing Core Facility for sequencing.

### HiC.

Frozen H1644 cell pellets were shipped to Active Motif Inc. for Hi-C service. Resulting libraries were submitted to the NIEHS Epigenomics and DNA Sequencing Core Facility for sequencing.

### MTT assay.

Media was changed to vehicle decidual media, and siNT or siZMIZ1 cells were grown until confluent, then passaged to 96 well dishes at 1,000 cells per well (H1644) or 5,000 cells/well (Ishikawa cells) and cultured in decidual media with vehicle or with 10 nM estradiol for 0–5 days. Viability was assessed with a Cell Proliferation Kit I (MTT; Roche) according to the manufacturer’s protocol.

### DNA synthesis assay.

Media was changed to vehicle decidual media, and siNT or siZMIZ1 H1644 cells were grown for 2 days. Media was changed to vehicle decidual media containing 10 μM EdU, cells were harvested after 24 hours, and EdU was detected using the Click-iT EdU Flow Cytometry Assay Kit (Thermo Fisher) according to the included protocol. The percentage of EdU positive cells was counted using the LSRFortessa cell counter (BD Biosciences).

### Mouse strains.

B6.129-Pgr<tm2(Cre)Lyd> (PgrCre) mice (42) were from our onsite breeding colony. C57BL/6-Zmiz1<tm1c(EUCOMM)Hmgu> (Zmiz1-flox) mice (21) were provided by Mark Chiang. PgrCre mice were intercrossed with Zmiz1-flox mice to produce mice with uterine *Zmiz1* deletion (PgrCrexZmiz1-flox; Zmiz1^d/d^). For genotyping, ear biopsies were sent to Transnetyx, Inc. Resulting Cre^+^ mice homozygous for Zmiz1-flox allele were generally normal and healthy.

### Breeding trial.

Individual Zmiz1^d/d^ females or their Zmiz1^f/f^ littermates were housed with a control male for 6 months. Dates and sizes of litters produced were recorded.

### Super ovulation.

Adult females (9–13 weeks old) were injected with pregnant mare serum gonadotrophin (Calbiochem; 3.25 IU per mouse) and 48 hours later with human chorionic gonadotrophin (Calbiochem; 5 IU per mouse). The following morning, oocytes in the oviducts were counted.

### Pregnancy stage–specific studies.

Adult females (8+ weeks old) were individually housed overnight with a male and checked for the presence of a copulatory plug in the morning to confirm mating, which is 0.5 dpc. Uterine tissue was collected from plug-positive females on days indicated in each experiment. For IHC analysis, tissue was fixed overnight in 4% paraformaldehyde then transferred to 70% ethanol and paraffin embedded. Embryos were flushed from uteri and oviducts of females collected on 3.5 dpc and counted. For progesterone levels, serum was sent to the Ligand Core Ligand Assay & Analysis Core at the University of Virginia. Prolactin levels were measured using the DuoSet ELISA Mouse Prolactin kit (R&D Systems).

### Estrous cycle monitoring.

Saline (10 μl, Sigma) vaginal washings were collected each morning for 2 weeks, stained with Giemsa stain and then evaluated for cycle stage.

### Decidual response in mice.

Ovaries were removed and mice were held for 10–14 days to allow endogenous hormones to clear. Mice were injected subcutaneously (sc) daily for 3 days with estradiol (Steraloids; 100 μl of 1 μg/ml estradiol in Sesame Oil; Sigma), followed by 2 days with no injections, then daily sc injections of progesterone (Steraloids) together with estradiol (100 μl of a solution containing 10 mg/ml progesterone and 67 ng/ml estradiol in sesame oil). On the afternoon of the third progesterone-plus-estrogen injection, approximately 6 hours after the injection, 50 μl of sesame oil was injected into the lumen of the uterine horn as a decidual stimulus to mimic embryo apposition. Daily progesterone-plus-estrogen injections continued until uterine tissue was collected 20 hours, 3 days, or 5 days after the decidual stimulus for histological, RNA, and protein analyses. In the instance of the collections 20 hours after decidual stimulus, EdU (100 μl of a 2 mg/ml solution in PBS; Thermo Fisher) was administered by intraperitoneal injection 18 hours after decidual stimulus and uterine tissue was collected 2 hours later.

### Estrogen response in mice.

Ovariectomized mice were held for 10–14 days to allow endogenous hormones to clear, then intraperitoneally injected with 100 μl of saline vehicle (Sigma) or saline containing 2.5 μg/ml of estradiol. Twenty-two hours later, EdU (100 μl of a 2 mg/ml solution in PBS; Thermo Fisher) was administered by intraperitoneal injection and uterine tissue was collected 2 hours later.

### Histological analysis of ZMIZ1 expression in human endometrium and endometriosis lesions.

Human endometrial tissues and endometriotic lesions were obtained from participants under a protocol approved by Baylor College of Medicine Institutional Review Board (IRB ID # H-52920 and H-52167) and Washington University in St. Louis School of Medicine Institutional Review Board (IRB ID # 201612127 and 201807160). The tissue biopsies were embedded in paraffin and sectioned (5 μm) for histological study as reported earlier (70). Briefly, sections were deparaffinized, rehydrated in ethanol gradient, and were boiled for 20 minutes in citrate buffer (Vector Laboratories Inc.) for antigen retrieval. Endogenous peroxidase activity was quenched using Bloxall (Vector Laboratories Inc.). Sections were then blocked with 2.5% goat serum in PBS for 1 hour (Vector Laboratories Inc.). After 3 washes in PBS, tissue sections were incubated overnight at 4°C in 2.5% goat serum containing the ZMIZ1 (1:100) primary antibody (Cell Signaling Technology, Cat. No. 89500). The next day, tissue sections were incubated for 1 hour with a biotinylated secondary antibody, washed, and treated with ABC reagent (Vector Laboratories Inc.) for 45 minutes. Immunoreactivity was developed with 3, 3′-diaminobenzidine (DAB) peroxidase substrate (Vector Laboratories Inc.), and sections were counter stained with hematoxylin. Eventually, sections were dehydrated and mounted in Permount histological mounting medium (Fisher Scientific).

### Immunostaining of mouse tissues.

Paraformaldehyde-fixed mouse tissue samples were embedded in paraffin and sectioned at 5 μm onto charged slides. Sections were deparaffinized and rehydrated then processed for 5–10 minutes in Antigen Decloaker Buffer (Biocare Medical) using the Biocare Medical Decloaking Chamber. For ZMIZ1 IHC, endogenous peroxidase was blocked using 5% hydrogen peroxide (Fisher Chemicals) for 10 minutes, then tissues were blocked in 10% Normal Goat Serum (Jackson Immunoresearch) diluted in wash buffer (50 mM Tris; (Lonza), 20 mM NaCl (Lonza) with 0.5% Tween 20 (Sigma), followed by blocking with the Avidin-Biotin Blocking kit (Vector Labs). Rabbit anti ZMIZ1 (Cell Signaling Technologies Catalog 89500) diluted 1:100 in blocking buffer was applied for 1 hour. After washing, sections were incubated with biotinylated anti Rabbit IgG (Vector Labs Catalog Ba-1000) diluted 1:500 in wash buffer, followed by Streptavidin, Peroxidase, R.T.U. (Vector Labs), detected with ImmPACT DAB Substrate Kit (Vector Labs) and counterstained with Hematoxylin (Epredia). Slides were dehydrated and mounted with Permount (Fisher).

In estrogen-treated samples, Ki67 was detected as previously described (71) using rabbit polyclonal anti-Ki67 (BD Pharminogen# 550609) at a 1:100 dilution. In decidual samples, Ki-67 was detected with the Vector ImmPRESS Anti-Rabbit Kit (Cat#MP-7401). Tissue sections were deparaffinized and rehydrated, then heat-induced epitope retrieval was performed using a citrate buffer (pH6.0, Biocare Medical) in the Decloaker pressure chamber for 15 minutes at 110°C. Endogenous peroxidase blocking was using 3% H_2_O_2_ for 15 minutes, then sections were blocked for 20 minutes with 2.5% normal horse serum (Vector ImmPRESS) followed by rabbit Ki-67 antibody (Cat# ab16667, Abcam) at a 1:200 dilution for 60 minutes at room temperature. The antigen-antibody complex was detected using anti-rabbit IgG HRP polymer (Vector ImmPRESS) and DAB (Dako).

For PGR, IHC tissue sections were deparaffinized and rehydrated, then heat-induced epitope retrieval was performed using a 10 mM citrate buffer solution, pH 6.0 (Biocare Medical) in the Decloaker pressure chamber for 15 minutes. Endogenous peroxidase was blocked using 3% H_2_O_2_, then sections were blocked for 20 minutes with 2.5% normal horse serum (Vector ImmPRESS) and incubated with Rabbit monoclonal Anti-Progesterone Receptor antibody (Cell Signaling Technology, Cat# 8757l) at a 1:1,000 dilution for one hour at room temperature; sections were incubated with Vector ImmPRESS anti-rabbit HRP Polymer (Vector Laboratories) for 30 minutes at room temperature. The antigen-antibody complex was visualized using DAB chromogen (DakoCytomation) for 6 minutes.

For ESR1 IHC, tissue was deparaffinized and rehydrated epitope retrieval was performed in a 10 mM citrate buffer solution, pH 6.0 (Biocare Medical,) in the Decloaker pressure chamber for 15 minutes at 110°C. Endogenous peroxidase was blocked using 3% H_2_O_2_; then, sections were blocked using Rodent Block M (Biocare Medical) for 20 minutes at room temperature. The sections were then incubated with rabbit polyclonal Anti-Estrogen Receptor α antibody (EMD Millipore Cat# 06-935) at a 1:2,000 dilution for one hour then incubated with Rabbit-on-Rodent HRP Polymer (Biocare Medical) for 30 minutes. The antigen-antibody complex was visualized using DAB chromagen (DakoCytomation) for 6 minutes at room temperature.

EdU was detected in deparaffinized uterine tissue sections using the Click-iT EdU Imaging Kit (Thermo Fisher) according to the included protocol.

For IFA colocalization of ESR1 and ZMIZ1, PFA fixed uterine tissue was dehydrated in progressively graded sucrose (10%–20%) and cryo-embedded in 10% sucrose+50% OCT (Fisher). Tissue sections were processed for 10 minutes in Antigen Decloaker Buffer, then washed with PBS+0.1% Triton X100 (Sigma) for 5 minutes and rinsed in wash buffer. Tissue was blocked for 30 minutes in 5% normal donkey serum in wash buffer, then incubated overnight at 4°C with sheep anti-ZMIZ1 (R&D Systems AF8107) diluted 1:200 and rabbit anti-ESR1 (Cell Signaling Technologies Cat#13258) diluted 1:500 in blocking buffer. After washing, anti-sheep Alexafluor 647 and anti-rabbit Alexafluor 568 (both Thermo) diluted 1:200 in block buffer were added for 30 minutes. Sections were washed, counterstained with DAPI, mounted with Prolong Gold (Thermo) and imaged using a Zeiss 780 Confocal microscope.

### RNA isolation and analysis.

Trizol (Thermo Fisher) was used to isolate RNA from either cells or uterine tissue according to the manufacturer’s protocol. Following chloroform extraction, RNA was prepared from the aqueous layer using the Direct-zol RNA Miniprep Kit (Zymo Research). For RT-PCR, cDNA was synthesized and analyzed using real time PCR as previously described (72). Primer sequences are listed in Supplemental Table 6.

### RNA-seq.

RNA was submitted to the NIEHS Epigenomics and DNA Sequencing Core Facility for RNA seq. Libraries were made using the Illumina Tru Seq Stranded mRNA kit and sequenced with the Illumina NovaSeq 6000. FASTQ files were imported into Partek Flow (Illumina), aligned to hg38 or mm10 using Bowtie 2, then quantified to Refseq genes and reads were normalized. Differentially expressed genes were found using ANOVA.

### Microarray.

For microarray analysis, RNA was submitted to the NIEHS Molecular Genomics Core Facility for hybridization using the Affymetrix MTA 1.0 chip (Thermo Fisher). Output data was analyzed using the Applied Biosystems Transcript Analysis Console (Thermo Fisher). Differentially expressed genes were uploaded to Ingenuity Pathway Analysis (Qiagen) for Core Analysis.

### ChIPseq analysis.

FASTQ files were imported into Partek Flow (Illumina) and aligned to hg38 using Bowtie2.

### HiC analysis.

Reads were aligned to hg38 and loops called as described previously (72).

### Western blot.

Uterine tissue was homogenized in 50 mM Tris (Lonza), 150 mM NaCl (Lonza), 1% Triton X100, 2.5 mg/ml Sodium Deoxycholate (Sigma) containing Phosphatase inhibitor cocktails 2&3 (Sigma) and Complete Protease Inhibitor (Roche) and concentration determined using the BCA Assay (Pierce). 25–50 μg of protein was separated on 10% Mini-Protean Precast Gels (Bio Rad) and transferred to nitrocellulose membrane using Trans-Blot Turbo Transfer System (Bio Rad) according to manufacturer’s protocols. Filters were blocked for 1 hour with 5% Blotto (Santa Cruz Biotechnology) dissolved in TBST (50 mM Tris 138 mM NaCl with 0.1% Tween 20; Sigma). Antibodies to PGR (Cell Signaling Technologies 8757 1:1000), ESR1 (Millipore 06-935 1:5000), ZMIZ1 (Cell Signaling Technologies 89500 1:1000), or GAPDH (Santa Cruz Biotechnologies sc-25778 1:5000) were diluted in blocking buffer and incubated with membranes overnight. After 3 washes with TBST, anti-rabbit IgG labeled with IR 800 or IR 680 (Licorbio) was diluted 1:10,000 in blocking buffer and incubated with membranes for 45 minutes. Bands were detected and quantified using the Licorbio Fc instrument with ImageStudio software.

### Statistics.

Significance was tested with GraphPad Prism software using 2-tailed t test or 2-way ANOVA as indicated in each figure legend. A *P* value less than 0.05 was considered significant.

### Study approval.

Human samples were obtained following protocols approved by the Institutional Review Boards of Michigan State University and Spectrum Health Medical System (Grand Rapids, MI IRB ID # LEGACY09-1213), Baylor College of Medicine Institutional Review Board (IRB ID # H-52920 and H-52167) or Washington University in St. Louis School of Medicine Institutional Review Board (IRB ID # 201612127 and 201807160). Prior to the participation, written informed consent was obtained from the patients. Animal studies were conducted according to an Animal Study Proposal approved by the NIEHS Animal Care and Use Committee.

### Data availability.

RNA-seq (GSE288879, GSE288883, GSE288994), ChIP-seq (GSE288877), HiC (GSE288872) and Microarray data (GSE288806) are available on GEO (GSE288995). Data values are available in the Supporting Data Values file.

## Author contributions

SCH, GVND, CD, JR, RK, and FJD designed research studies. SCH, FO, MY, GVND, and CD conducted experiments and acquired data. SCH, FO, RMM, GVND, RK, and CJW analyzed data. MYC, YS, RK, ATF, and JPL provided reagents. SCH and FJD wrote the manuscript.

## Funding support

This work is the result of NIH funding, in whole or in part, and is subject to the NIH Public Access Policy. Through acceptance of this federal funding, the NIH has been given a right to make the work publicly available in PubMed Central.

- Postdoctoral Research Associate (PRAT) fellowship from the National Institute of General Medical Sciences (NIGMS) FI2GM154603 (RMM).
- Lalor Foundation fellowship (RMM).
- NIH/NICHD R01 HD-042311 (to JPL).
- NICHD R01HD104813 (to RK).
- Intramural Research Program of the NIH.
- National Institute of Environmental Health Sciences, project Z1AES103311-01.

## Supplementary Material

Supplemental data

Unedited blot and gel images

Supplemental table 1

Supplemental table 2

Supplemental table 3

Supplemental table 4

Supplemental table 5

Supplemental table 6

Supporting data values

## Figures and Tables

**Table 1:**
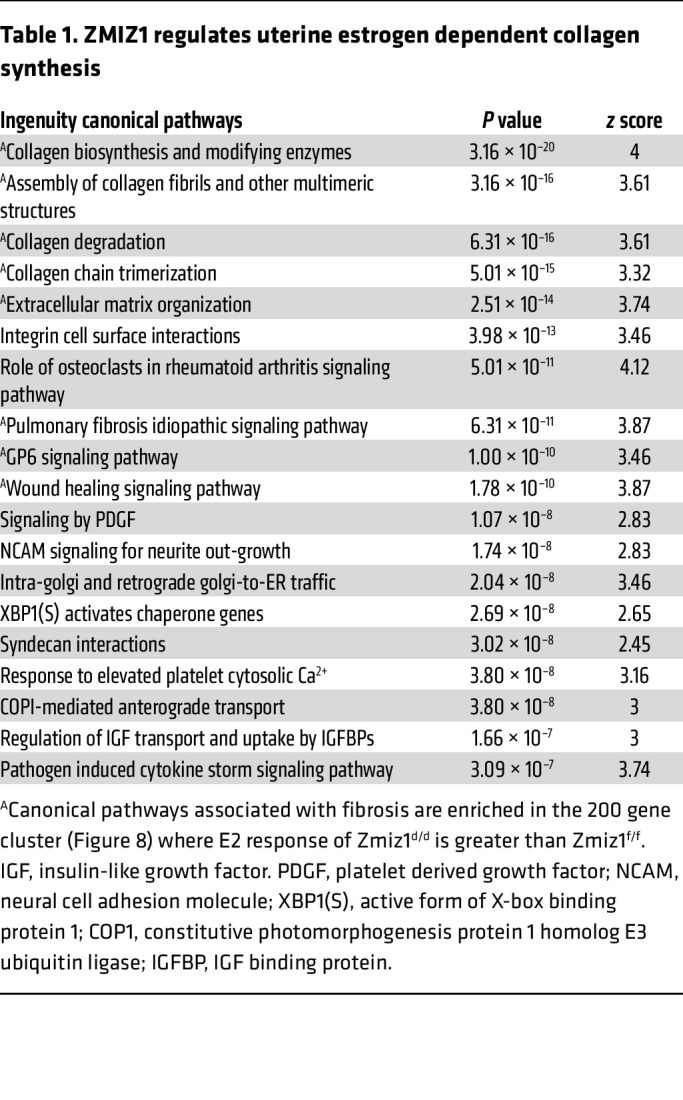
ZMIZ1 regulates uterine estrogen dependent collagen synthesis

**Table 2:**
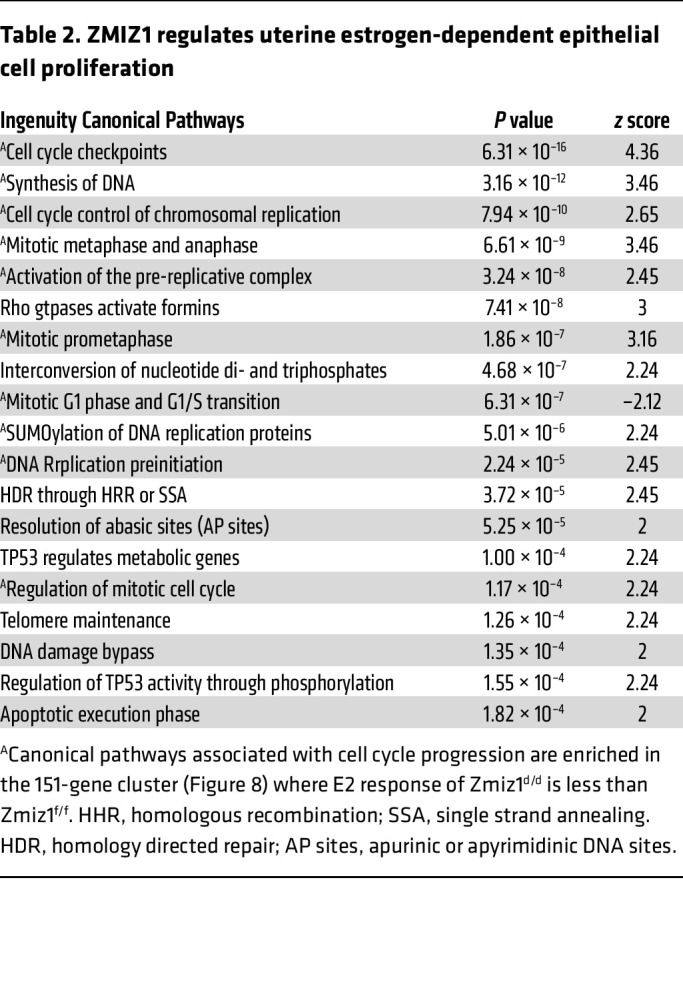
ZMIZ1 regulates uterine estrogen-dependent epithelial cell proliferation

## Version 1 12/01/2025

Electronic publication
