# Supplementary material for: Transcriptional coregulator ZMIZ1 modulates estrogen responses that are essential for healthy endometrial function
Source: J Clin Invest. 2025 Dec 1;135(23):e193212. doi: 10.1172/JCI193212 (PMC12646675; doi:10.1172/JCI193212)

Transcriptional co-regulator ZMIZ1 modulates estrogen responses that are essential for healthy endometrial function.

Supplemental Figure Legends

Supplemental Figure 1 ZMIZ1 in Human Endometrium

A. Chromatin landscapes near mouse (top panel) and human (bottom panel) *ZMIZ1* genes. Mouse tracks include ESR1 (blue) and Histone H3 K27 acetylation (K27Ac, red) ChIPSeq, and loops called from previously published HiC data (1). Potential enhancers (ESR1 and K27Ac peaks) are indicated with dashed outlines; previously described superenhancers (SE) are highlighted in yellow with each one's rank shown below it. Human tracks include proliferative endometrium ESR1 ChipSeq (blue) (2) and K27Ac (red) and HiC loops from cultured H1644 hESC.

B. *ZMIZ1* transcript expression level in each endometrial cell type and at multiple menstrual cycle stages from scRNAseq dataset described in Figure 1B.

C. RT_PCR of *ZMIZ1* levels in THESC cells following 3 days of culture with vehicle (V) or decidualized with EPC or omission of estrogen (PC). N=3 each. ****,***,** p<0.0001,0.001,0.01, respectively, by ANOVA.

D. Effect of siNT or siZMIZ1 targeting on Ishikawa cells. Left: MTT A595 signal on day 2 after estrogen (E2) treatment was initiated, calculated as % that on the day E2 was added (d0). n=6 for each condition. Center and right: RT-PCR for *ZMIZ1*, *PGR*, or *GREB1* in Ishikawa cells targeted with siNT or siZMIZ1 and then treated for 2 days with ethanol vehicle (V) or with 10 nM estradiol (E2). n=3 for all conditions. **,***, **** p< 0.01,0.001, 0.0001, respectively, by two way ANOVA.

Supplemental Figure 2 Analysis of targeting *Zmiz1* in mouse uterine tissue.

A. ZMIZ1 IHC of uterine cross sections taken from ovariectomized Zmiz1$^{f/f}$ or Zmiz1$^{d/d}$ mice injected with estrogen for 2 days, rested for 2 days and then injected with progesterone for 4 days. Scale bars=250 μm (top) or 200 μm (bottom).
B. ZMIZ1 Western blot of total uterine proteins from Zmiz1$^{f/f}$ (n=2) and Zmiz1$^{d/d}$ (n=3) mice. The arrow indicates the 120 kilodalton ZMIZ1 band.
C. RT-PCR for *Zmiz1* of uterine RNA from Zmiz1$^{f/f}$ or Zmiz1$^{d/d}$ mice treated with V or estradiol (E2) for 2 or 24 hours (h). For Zmiz1$^{f/f}$ V n=4 for all treatments, for Zmiz1$^{d/d}$ V n=5, Zmiz1$^{d/d}$ E2, 2h and E2, 24h n=6. **** p< 0.0001, by 2-way ANOVA.

Supplemental Figure 3 Impact of *Zmiz1* deletion on female mouse reproductive tract.

A. Pups produced during 6 months of continuous breeding of mice with uterine *Zmiz1* deletion (Zmiz1$^{d/d}$; n=6; **** p<0.0001 by unpaired t-test) compared to control (Zmiz1$^{f/f}$ n=6).

B. Representative uterine histology and analysis from aged (27-30 weeks old; scale bar=200 μm) or young (7-14 weeks old) Zmiz1$^{f/f}$ or Zmiz1$^{d/d}$ mice stained with H&E (scale bar=250 μm) or Masson's trichrome (scale bar=300 μm).

C. Estrous cyclicity evaluation of Zmiz1$^{d/d}$ females and Zmiz1$^{f/f}$ littermates using vaginal washes. P=proestrus E=estrus M=metestrus D=diestrus.

D. Evaluation of ovary function of Zmiz1$^{d/d}$ at 3.5 dpc or after superovulation. Graphs show number of oocytes collected following superovulation (n=4 Zmiz1$^{f/f}$, 8 Zmiz1$^{d/d}$; **=p<0.01 by unpaired t-test), embryos flushed from uterus at 3.5 dpc (n=17 Zmiz1$^{f/f}$, 15 Zmiz1$^{d/d}$ ns=non-significant by unpaired t-test), serum prolactin (n=7 Zmiz1$^{f/f}$, 8 Zmiz1$^{d/d}$ ns=non-

significant by unpaired t-test) or progesterone (n=5 Zmiz1$^{f/f}$, 4 Zmiz1$^{d/d}$ (from mice with embryos in uterus) *=p<0.05 by unpaired t-test) at 3.5 dpc. H&E sections of Zmiz1$^{f/f}$ or Zmiz1$^{d/d}$ ovaries from 3.5 dpc (scale bars Zmiz1$^{f/f}$=500 μm Zmiz1$^{d/d}$=1000μm) or superovulation (scale bar=300μm).

Supplemental Figure 4 *Zmiz1* deletion impairs decidual response.

A. RT-PCR of uterine RNA from decidualized Zmiz1$^{f/f}$ or Zmiz1$^{d/d}$ 3 days after intraluminal (il) oil injection (+) compared to un-injected uterine horn (-) showing *Bmp2,* the decidual prolactin *Prl8a2* and *Zmiz1*. For all samples n=4; *,** p<0.05, 0.01, respectively, by two-way ANOVA.
B. Weights of uterine horns from Zmiz1$^{d/d}$ mice and control littermates (Zmiz1$^{f/f}$) 3 days after il oil injection. For Zmiz1$^{d/d}$ and Zmiz1$^{f/f}$ littermates n=4; *p<0.05, by unpaired t-test.
C. H&E stained uterine sections from Zmiz1$^{d/d}$ or Zmiz1$^{f/f}$ littermates 3 days after intraluminal (il) oil injection (+) compared to un-injected uterine horn (-). Scale bars=300μm or 250 μm. Circled area highlights decidualized cells.
D. PGR or ESR1 Western blot of uterine proteins and ESR1 IHC from samples 20h after il oil injection (+) or un-injected controls (-). PGR-B and PGR-A are B and A isoforms of PGR, respectively. GAPDH is the loading control. Graphs show quantification of signal in each band (PGR or ESR1 band normalized to GAPDH band). For all conditions n=3. * p<0.05 by 2-way ANOVA. Scale bar=200μm.

Supplemental Figure 5 Zmiz1 Regulates Genes Driving Decidual Responses

A. Heat map showing the differentially expressed genes in Zmiz1$^{f/f}$ and Zmiz1$^{d/d}$ uterine RNA 20 h following il oil injection (+) or in non-injected controls (-).

B. Pathway summary shows the impact of il oil injection (+ vs -) in Zmiz1$^{f/f}$ or Zmiz1$^{d/d}$ on activity of pathways impacting the cell cycle. The heatmap shows the relative fold-changes (+ vs – il oil injection) of genes in the cell cycle checkpoint pathway.
C. RT-PCR of uterine RNA from Zmiz1$^{f/f}$ or Zmiz1$^{d/d}$ mice without (–) or 20 hours following il oil injection (+) of cell cycle genes *Ki67*, *Mcm2*, *Mcm5*, *Foxm1*, *Ccna2*, *Ccnb1*, *Ccnb2, Cdc2a* and *Zmiz1*. For Zmiz1$^{f/f}$ -, n=4; Zmiz1$^{f/f}$ +, n=4-6; Zmiz1$^{d/d}$ –, n=5; Zmiz1$^{d/d}$ +, n=3. *,**,***,**** p<0.05, 0.01, 0.001, 0.0001 by 2-way ANOVA.

Supplemental Figure 6 *Zmiz1* deletion enhances collagen synthesis and inhibits proliferation associated genes

A. RT-PCR of uterine RNA from Zmiz1$^{f/f}$ or Zmiz1$^{d/d}$ mice of collagen subunits *Col1a1*, *Col5a2*, *Col6a1*, and *Col6a3*. For Zmiz1$^{f/f}$ V n=9, Zmiz1$^{f/f}$ E24h n=10, Zmiz1$^{d/d}$ V n=8, Zmiz1$^{d/d}$ E24h n=-9. **,***,**** p<0.01, 0.001, 0.0001, by 2-way ANOVA.
B. Representative sections showing Masson's trichrome staining of ovariecomized Zmiz1$^{f/f}$ (n=3) or Zmiz1$^{d/d}$ (n=3) uterus treated for 24h with estradiol (E2). There is no apparent difference between the samples.
C. Heatmap of fold changes (E24h vs. V) of genes in the DNA synthesis pathway in Zmiz1$^{f/f}$ and Zmiz1$^{d/d}$ mice from analysis in Table 1. RT-PCR of uterine RNA from Zmiz1$^{f/f}$ or Zmiz1$^{d/d}$ mice of *Foxm1*, *Mcm2*, *Mcm5*, *Ccna2*, *Ccnb1*, *Ccnb2* and *Cdc2a*. For Zmiz1$^{f/f}$ V n=4-9, Zmiz1$^{f/f}$ E24h n=5-10, Zmiz1$^{d/d}$ V n=3-8, Zmiz1$^{d/d}$ E24h n=5-9. *,**,**** p<0.05, 0.01, 0.0001, ns=not significant by 2-way ANOVA.
D. Graphical summary of some of the top enriched signals, functions and pathways following IPA Core Analysis of DEG from RNAseq analysis of Zmiz1$^{d/d}$ vs Zmiz1$^{f/f}$ RNA 24h after estradiol (E24h) injection. Positive or negative z-score suggests Zmiz1 deletion increases or decreases signaling, respectively.

Supplemental Figure 7 Co-localization of ZMIZ1 and ESR1 in Uterine Cells

A. IFA of Zmiz1$^{f/f}$ or Zmiz1$^{d/d}$ uterine sections for ZMIZ1 (green) or ESR1 (red) in uterine tissue from mice treated as in Figure 7A, without il oil decidual stimulus, showing localization in nuclei of the same cells. DAPI (blue). Scale bar=10 μm.
B. IFA of Zmiz1$^{f/f}$ or Zmiz1$^{d/d}$ uterine sections for ZMIZ1 (green) or ESR1 (red) in uterine tissue from mice treated with E2 for 24h showing ZMIZ1 and ESR1 localization in nuclei of the same cells. Scale bar=10 μm.

References

1. Hewitt SC, Grimm SA, Wu SP, DeMayo FJ, and Korach KS. Estrogen receptor alpha (ERalpha)-binding super-enhancers drive key mediators that control uterine estrogen responses in mice. *The Journal of biological chemistry.* 2020;295(25):8387-400.
2. Hewitt SC, Wu SP, Wang T, Ray M, Brolinson M, Young SL, et al. The Estrogen Receptor alpha Cistrome in Human Endometrium and Epithelial Organoids. *Endocrinology.* 2022;163(9):2022.04.19.488787.

FS1

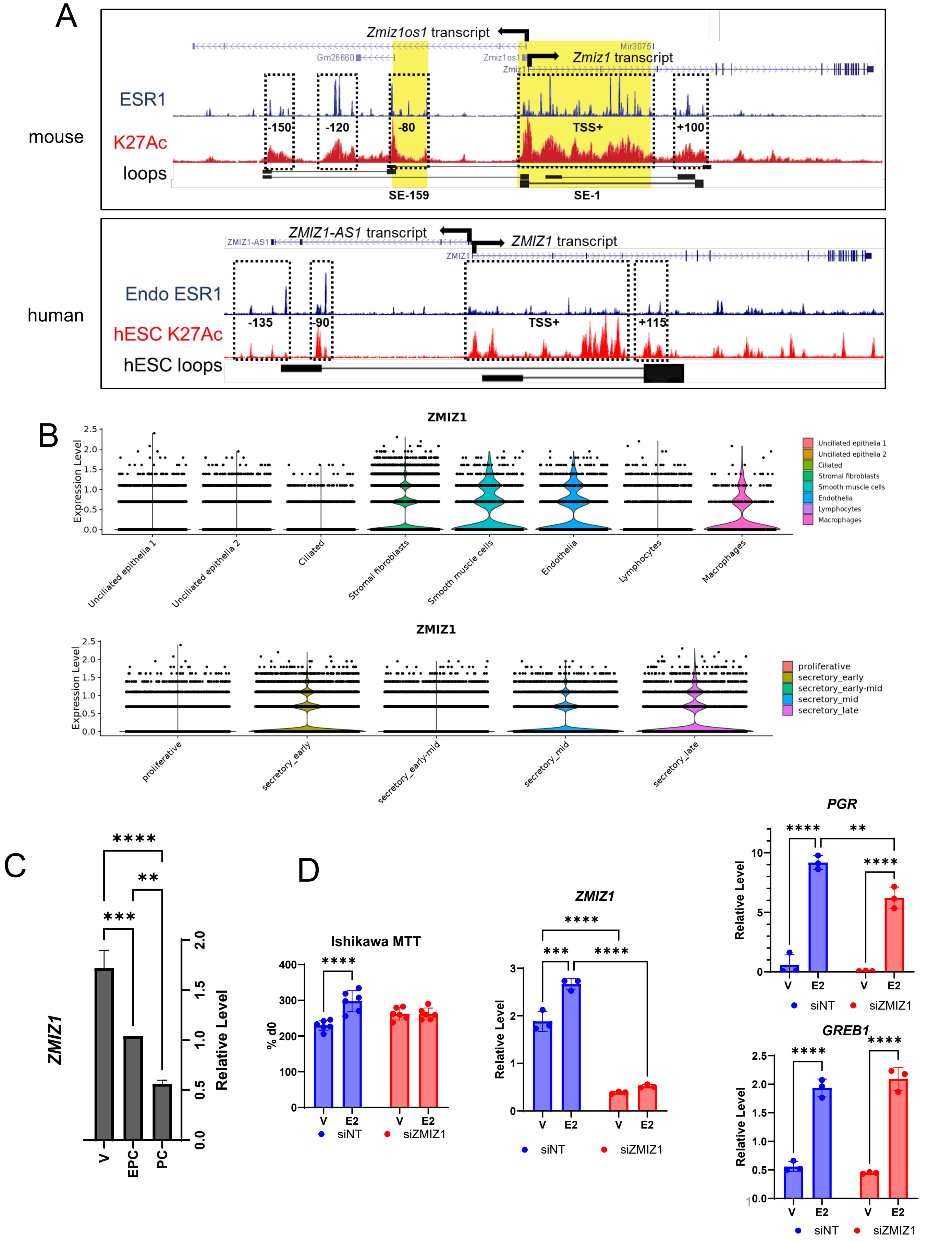

A
Zmiz1os1 transcript
Zmiz1 transcript
Gm26660
Zmiz1os1
Zmiz1
Mir3075
mouse
ESR1
K27Ac
loops
-150
-120
-80
TSS+
+100
SE-159
SE-1
human
ZMIZ1-AS1 transcript
ZMIZ1 transcript
ZMIZ1-AS1
ZMIZ1
Endo ESR1
hESC K27Ac
hESC loops
-135
-90
TSS+
+115
B
ZMIZ1
Expression Level
Unciliated epithelia 1
Unciliated epithelia 2
Ciliated
Stromal fibroblasts
Smooth muscle cells
Endothelia
Lymphocytes
Macrophages
proliferative
secretory_early
secretory_early-mid
secretory_mid
secretory_late
C
ZMIZ1
Relative Level
V
EPC
PC
D
Ishikawa MTT
% d0
PGR
GREB1
Relative Level
V
E2
siNT
siZMIZ1

FS2

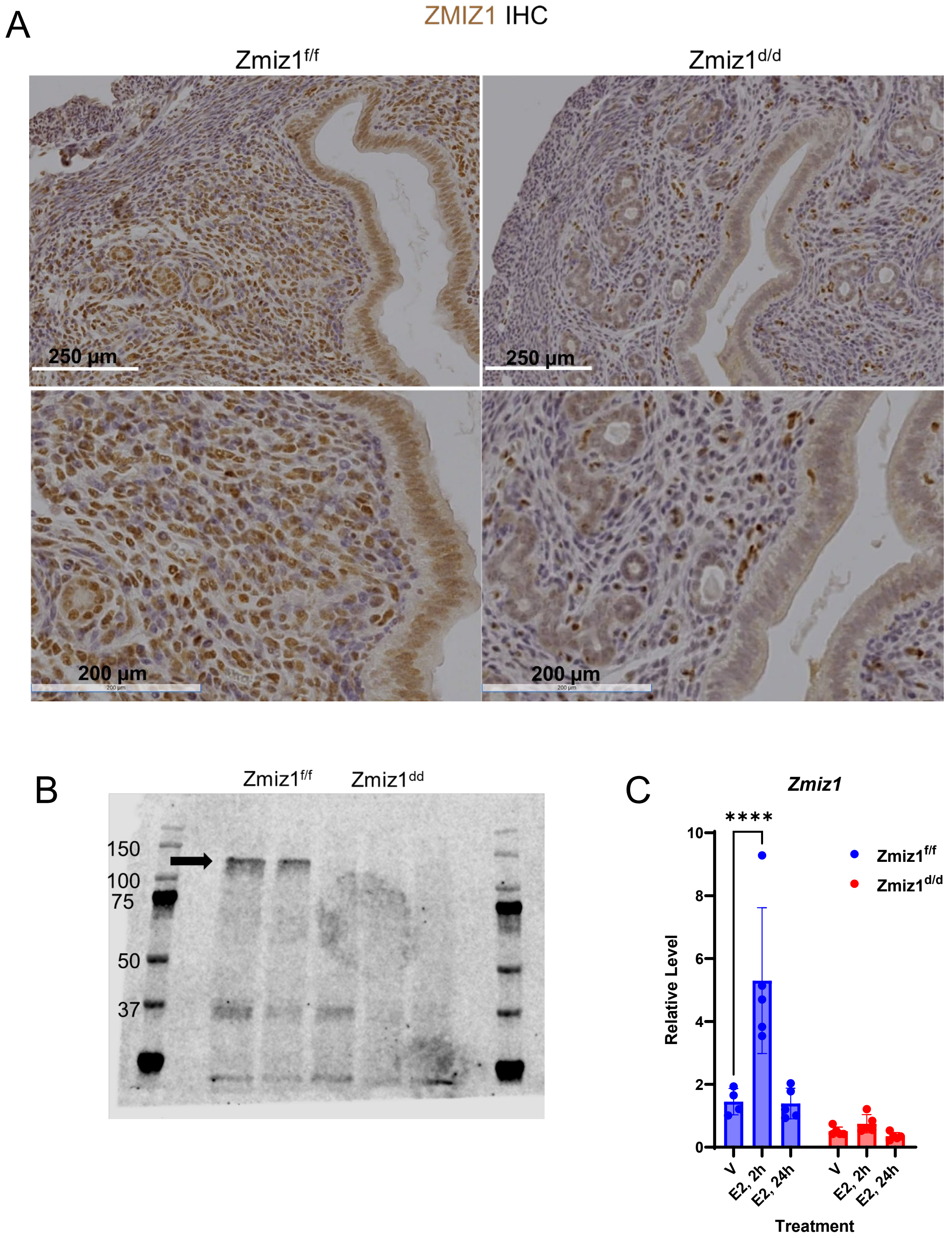
A
ZMIZ1 IHC
Zmiz1f/f
Zmiz1d/d
250 µm
250 µm
200 µm
200 µm
B
Zmiz1f/f
Zmiz1dd
150
100
75
50
37
C
Zmiz1
****
Relative Level
0
2
4
6
8
10
Zmiz1f/f
Zmiz1d/d
V
E2, 2h
E2, 24h
V
E2, 2h
E2, 24h
Treatment

Fig S3

A

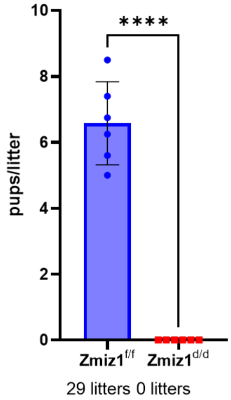

B

Sample Group	27-30 weeks old Zmiz1$^{f/f}$	27-30 weeks old Zmiz1$^{d/d}$	7-14 weeks old Zmiz1$^{f/f}$	7-14 weeks old Zmiz1$^{d/d}$
Atypical Glands	0/6	6/8	0/13	1/11 (mild)
Collagen Deposition	2/6	8/8	0/13	0/11

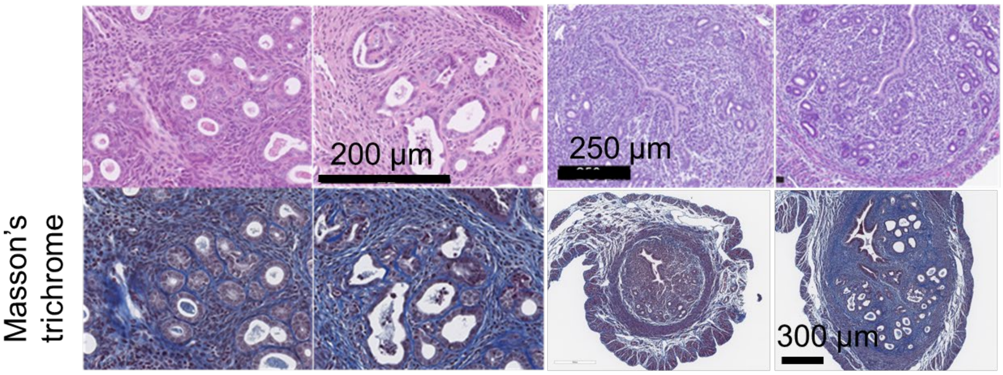

C

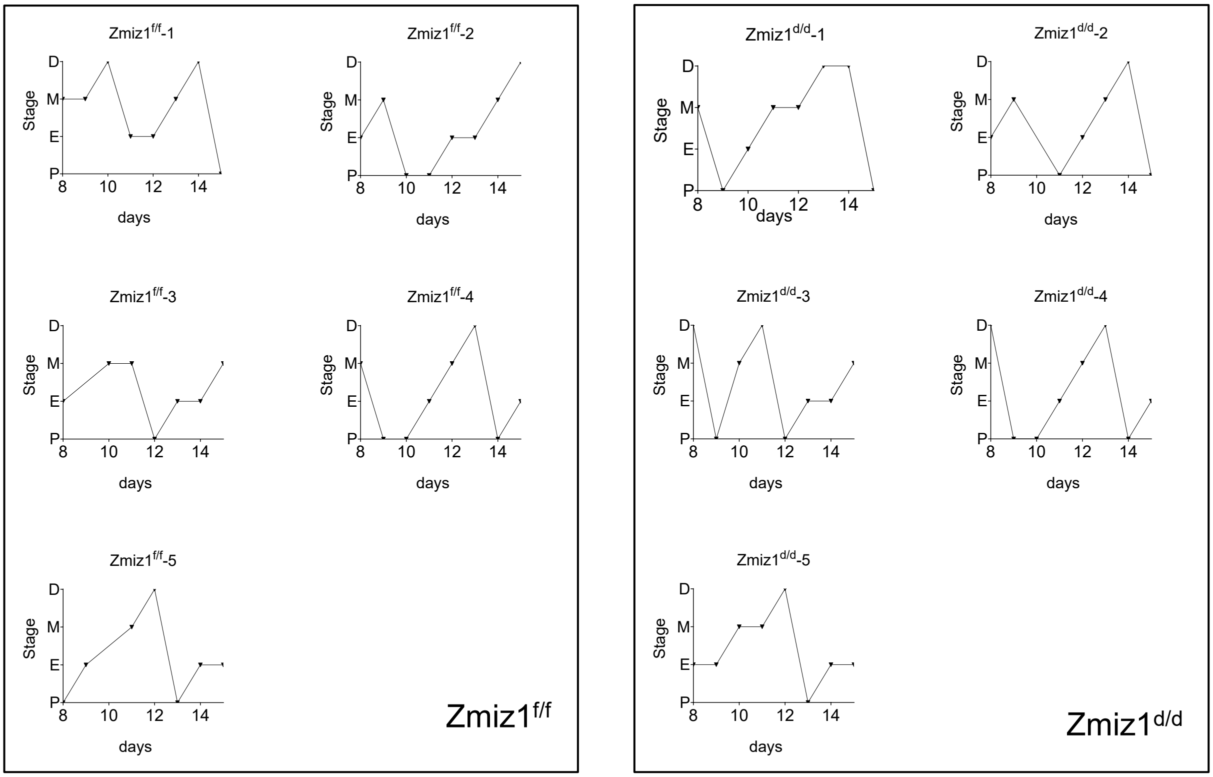

D

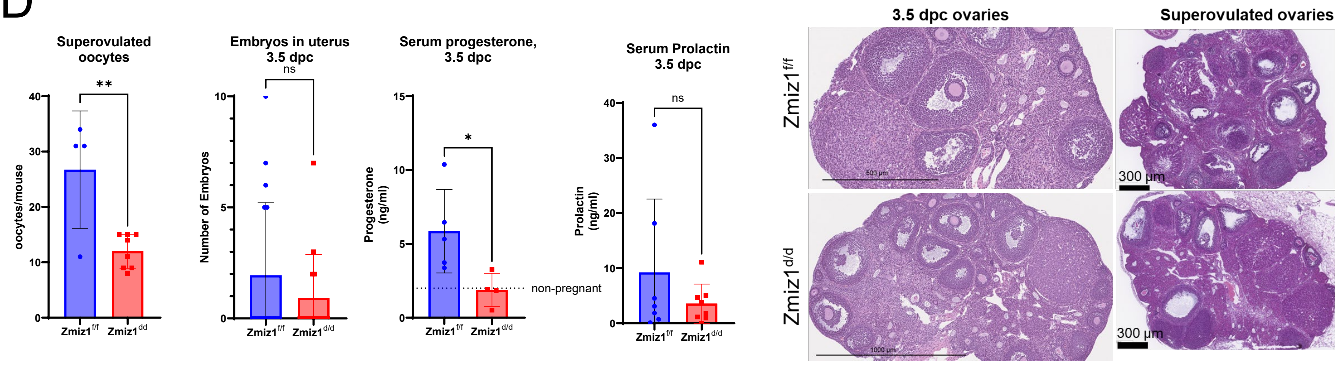

FS4

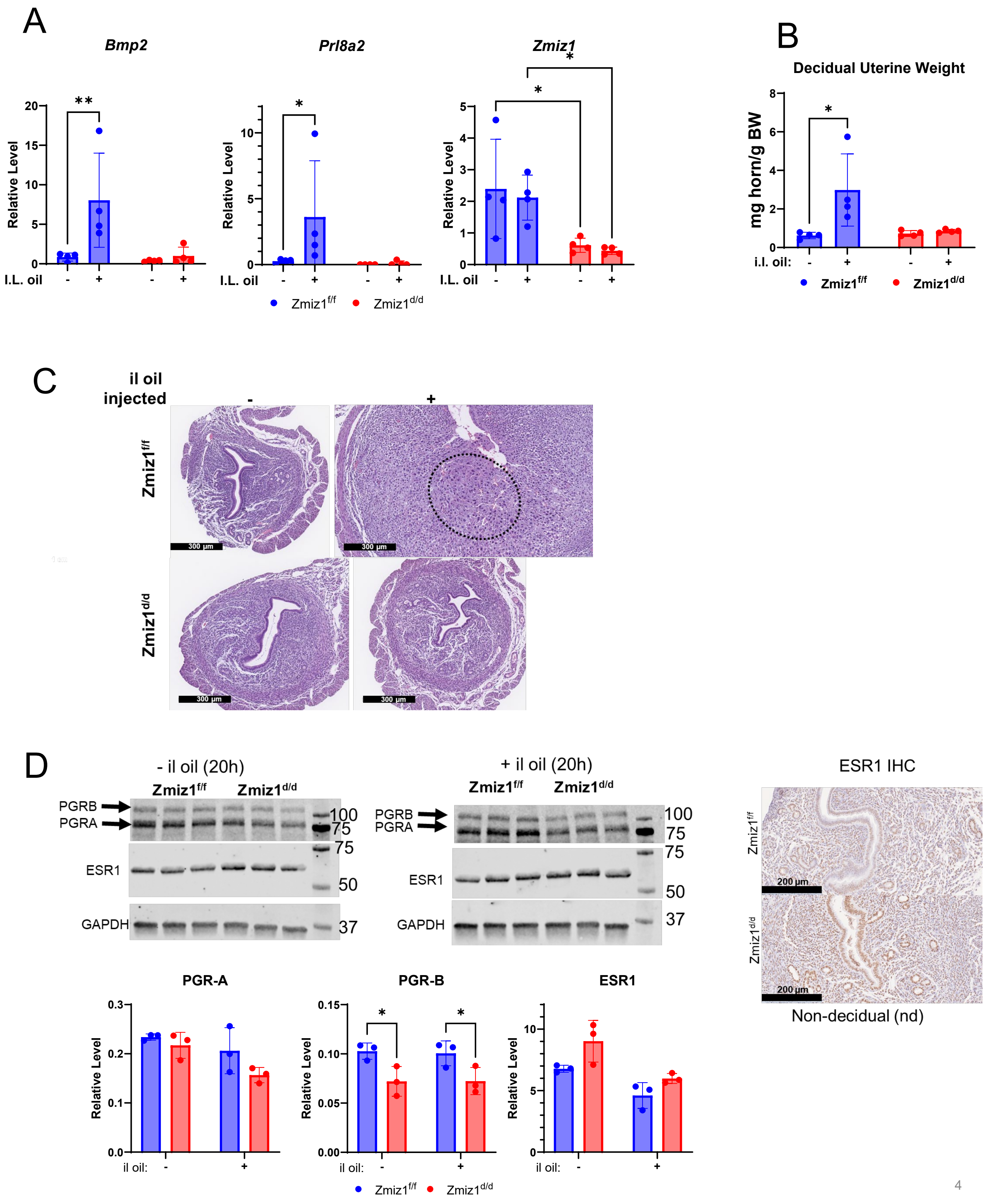
A
Bmp2
Prl8a2
Zmiz1
Relative Level
I.L. oil
Zmiz1f/f
Zmiz1d/d
B
Decidual Uterine Weight
mg horn/g BW
i.l. oil:
Zmiz1f/f
Zmiz1d/d
C
il oil injected
Zmiz1f/f
Zmiz1d/d
300 μm
D
- il oil (20h)
+ il oil (20h)
Zmiz1f/f
Zmiz1d/d
PGRB
PGRA
ESR1
GAPDH
100
75
50
37
ESR1 IHC
Zmiz1f/f
Zmiz1d/d
200 μm
Non-decidual (nd)
PGR-A
PGR-B
ESR1
Relative Level
il oil:
Zmiz1f/f
Zmiz1d/d

FS5

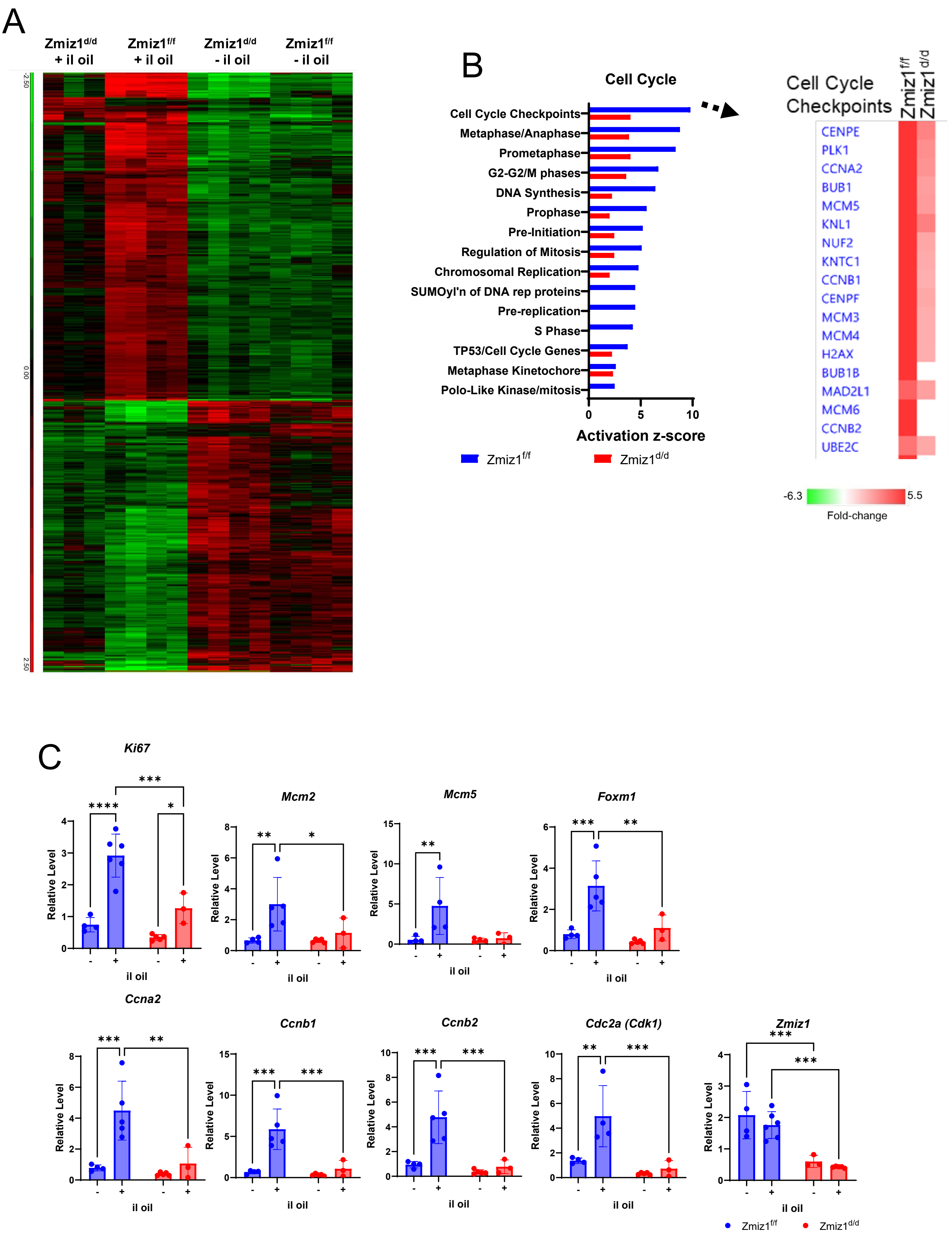
A
Zmiz1d/d + il oil
Zmiz1f/f + il oil
Zmiz1d/d - il oil
Zmiz1f/f - il oil
-2.50
0.00
2.50
B
Cell Cycle
Cell Cycle Checkpoints
Metaphase/Anaphase
Prometaphase
G2-G2/M phases
DNA Synthesis
Prophase
Pre-Initiation
Regulation of Mitosis
Chromosomal Replication
SUMOyl'n of DNA rep proteins
Pre-replication
S Phase
TP53/Cell Cycle Genes
Metaphase Kinetochore
Polo-Like Kinase/mitosis
0
5
10
Activation z-score
Zmiz1f/f
Zmiz1d/d
Cell Cycle Checkpoints
Zmiz1 f/f
Zmiz1 d/d
CENPE
PLK1
CCNA2
BUB1
MCM5
KNL1
NUF2
KNTC1
CCNB1
CENPF
MCM3
MCM4
H2AX
BUB1B
MAD2L1
MCM6
CCNB2
UBE2C
-6.3
5.5
Fold-change
C
Ki67
Mcm2
Mcm5
Foxm1
Ccna2
Ccnb1
Ccnb2
Cdc2a (Cdk1)
Zmiz1
Relative Level
il oil
Zmiz1f/f
Zmiz1d/d

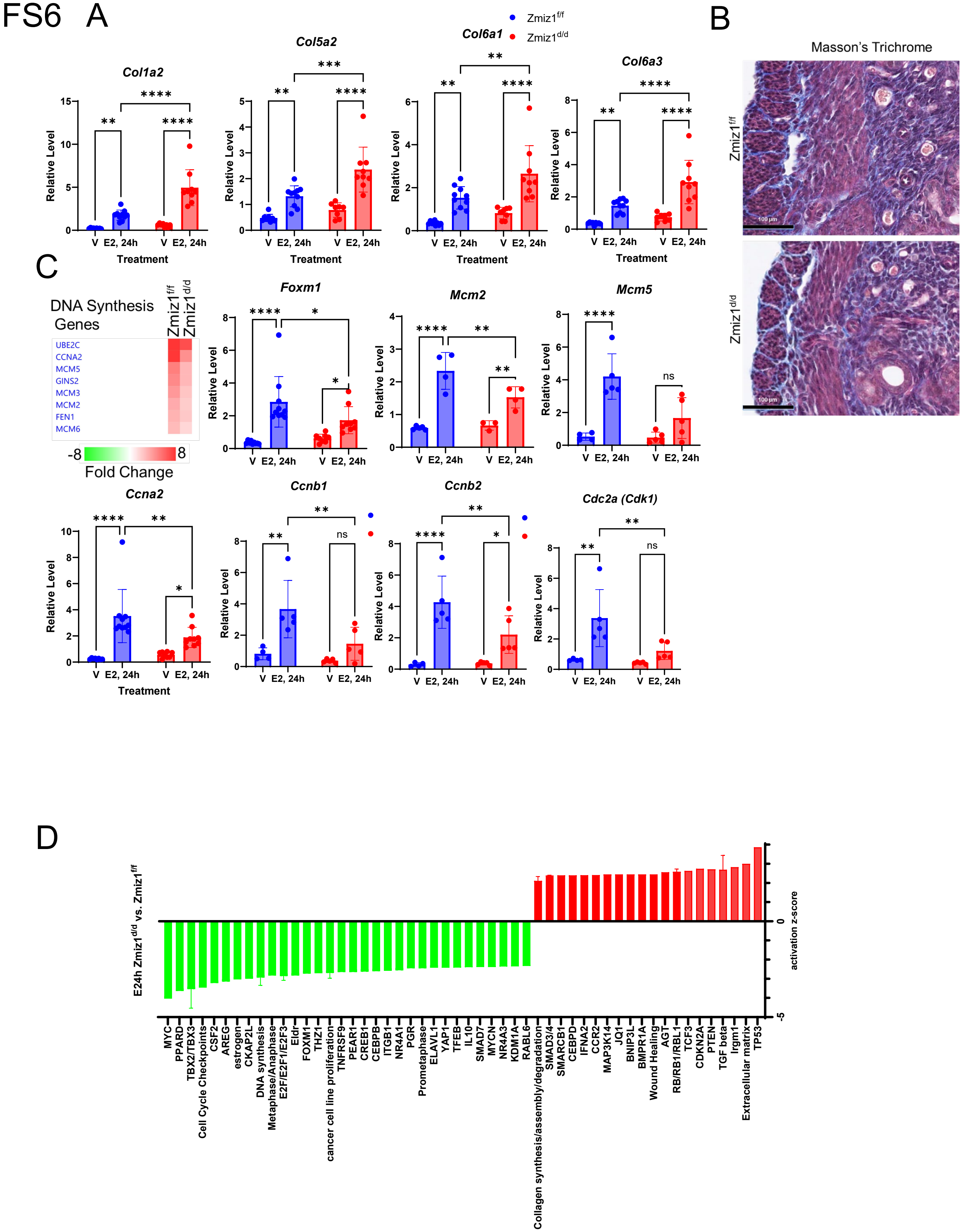
FS6
A
Zmiz1f/f
Zmiz1d/d
Col1a2
Col5a2
Col6a1
Col6a3
Relative Level
V
E2, 24h
Treatment
B
Masson's Trichrome
Zmiz1f/f
Zmiz1d/d
100 µm
C
DNA Synthesis Genes
UBE2C
CCNA2
MCM5
GINS2
MCM3
MCM2
FEN1
MCM6
-8
8
Fold Change
Foxm1
Mcm2
Mcm5
Ccna2
Ccnb1
Ccnb2
Cdc2a (Cdk1)
ns
D
E24h Zmiz1d/d vs. Zmiz1f/f
activation z-score
MYC
PPARD
TBX2/TBX3
Cell Cycle Checkpoints
CSF2
AREG
estrogen
CKAP2L
DNA synthesis
Metaphase/Anaphase
E2F/E2F1/E2F3
Eldr
FOXM1
THZ1
cancer cell line proliferation
TNFRSF9
PEAR1
CREB1
CEBPB
ITGB1
NR4A1
PGR
Prometaphase
ELAVL1
YAP1
TFEB
IL10
SMAD7
MYCN
NR4A3
KDM1A
RABL6
Collagen synthesis/assembly/degradation
SMAD3/4
SMARCB1
CEBPD
IFNA2
CCR2
MAP3K14
JQ1
BNIP3L
BMPR1A
Wound Healing
AGT
RB/RB1/RBL1
TCF3
CDKN2A
PTEN
TGF beta
Irgm1
Extracellular matrix
TP53

FS7

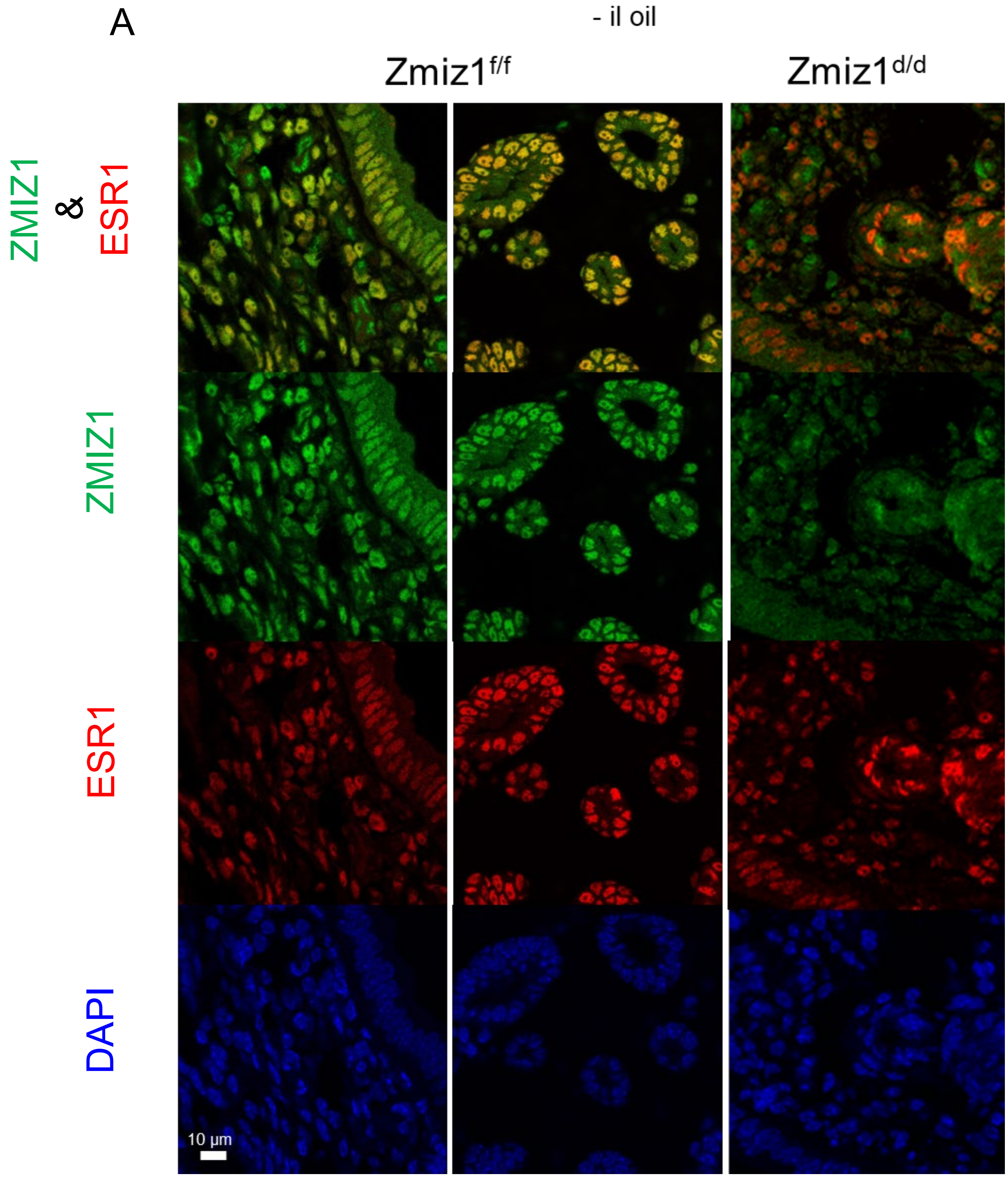

Fig S7

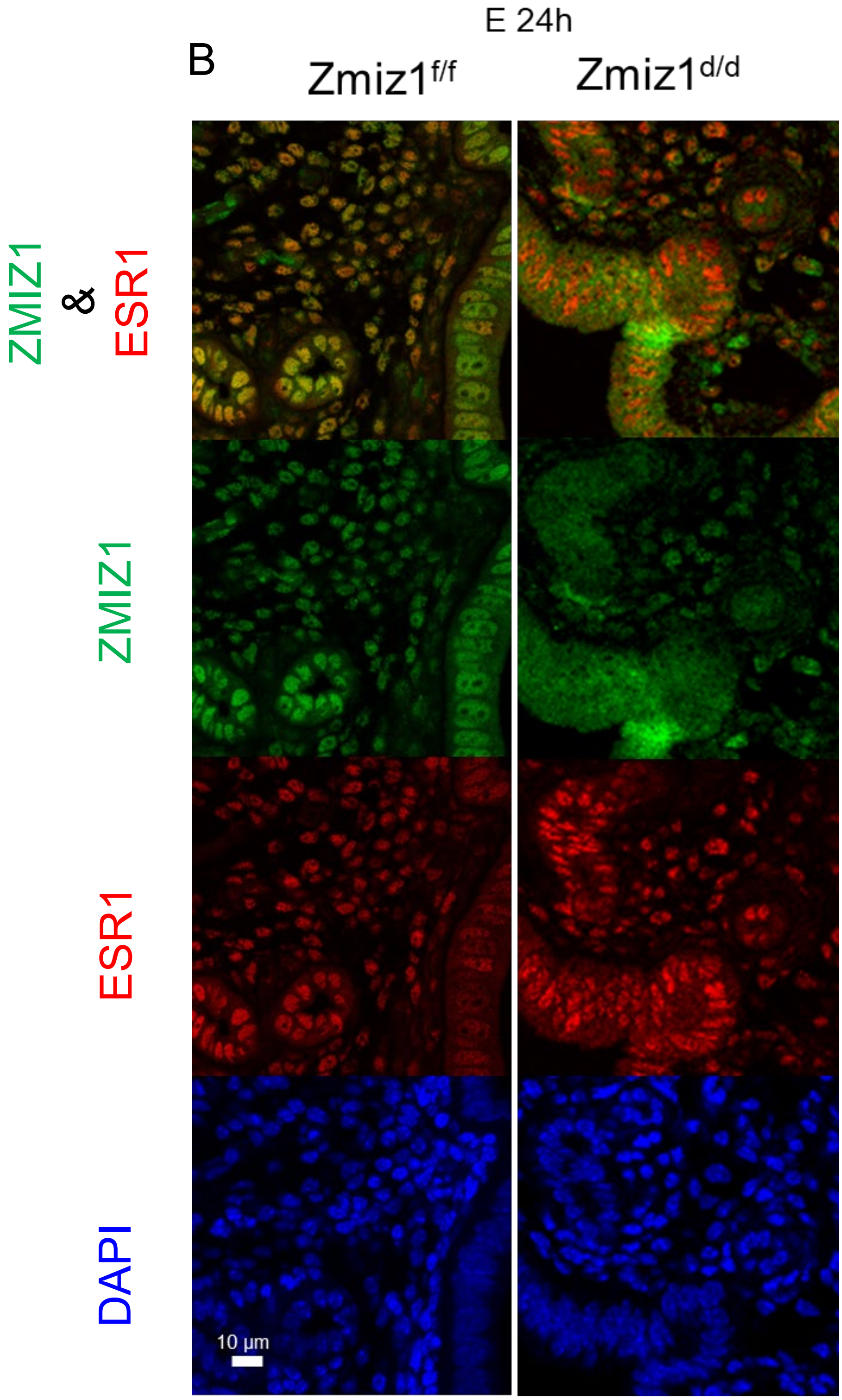

Supplement: Supplemental data [file jci-135-193212-s262.pdf]
