# Supplementary figures and images for: Transcriptional coregulator ZMIZ1 modulates estrogen responses that are essential for healthy endometrial function
Source: J Clin Invest. 2025 Dec 1;135(23):e193212. doi: 10.1172/JCI193212 (PMC12646675; doi:10.1172/JCI193212)

## S4D PGR

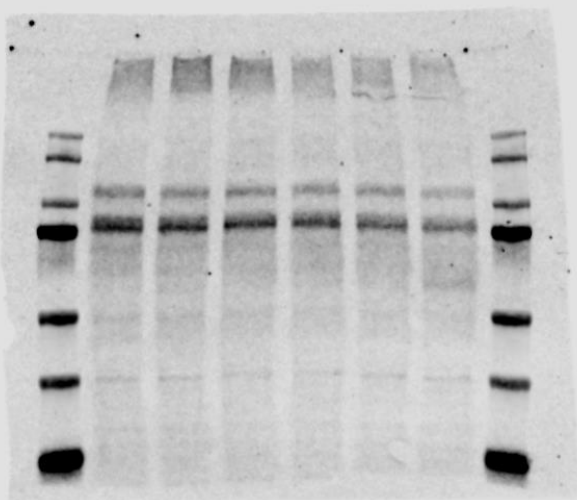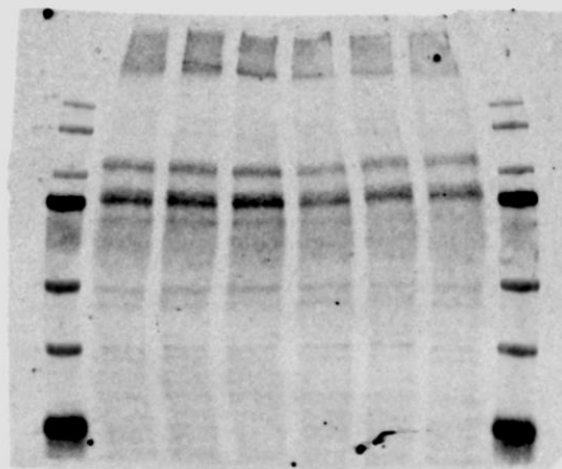

S4D ESR1

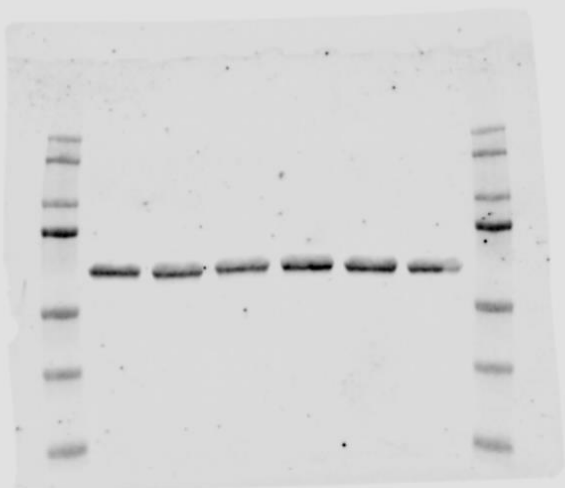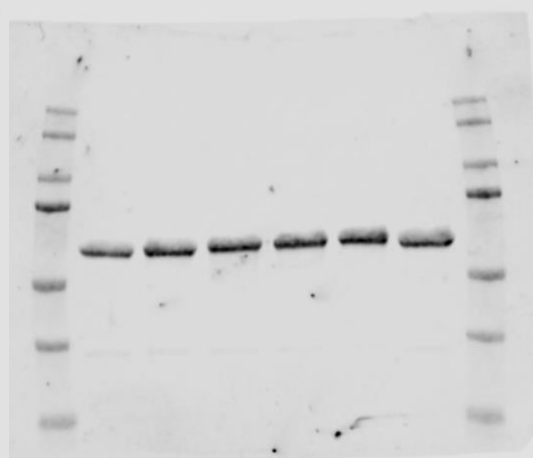

## S4D GAPDH

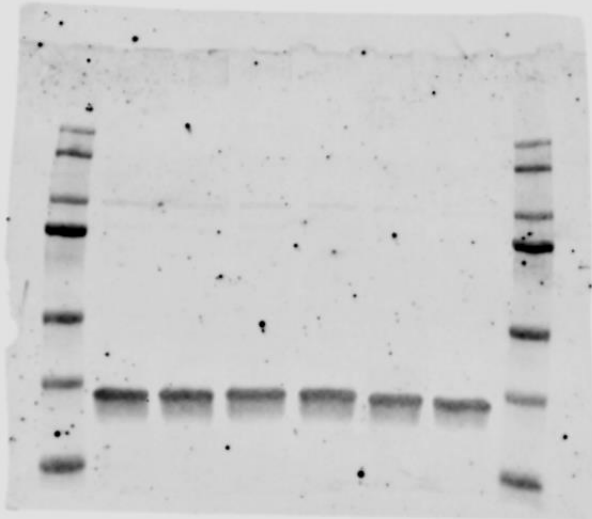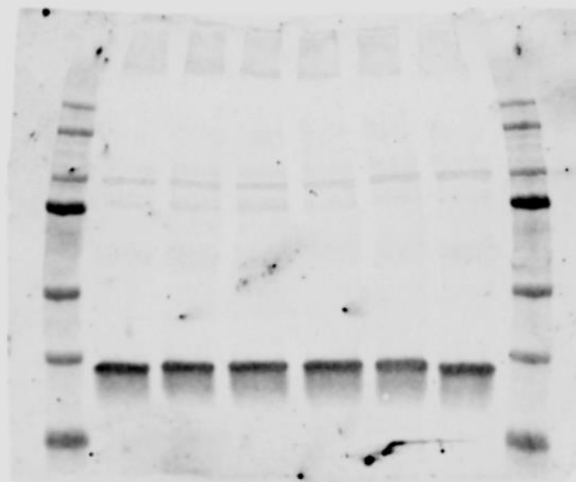

Supplement: Unedited blot and gel images [file jci-135-193212-s263.pdf]
